# Shp1 Loss Enhances Macrophage Effector Function and Promotes Anti-Tumor Immunity

**DOI:** 10.3389/fimmu.2020.576310

**Published:** 2020-09-29

**Authors:** Darienne R. Myers, Clare L. Abram, David Wildes, Amira Belwafa, Alia M. N. Welsh, Christopher J. Schulze, Tiffany J. Choy, Tram Nguyen, Neil Omaque, Yongmei Hu, Mallika Singh, Rich Hansen, Mark A. Goldsmith, Elsa Quintana, Jacqueline A. M. Smith, Clifford A. Lowell

**Affiliations:** ^1^Revolution Medicines, Inc., Redwood City, CA, United States; ^2^Department of Laboratory Medicine, University of California, San Francisco, San Francisco, CA, United States

**Keywords:** tyrosine phosphatase, phagocytosis, PTPN6, immuno-oncology, SIRPα, inflammation

## Abstract

Shp1, encoded by the gene *Ptpn6*, is a protein tyrosine phosphatase that transduces inhibitory signals downstream of immunoreceptors in many immune cell types. Blocking Shp1 activity represents an exciting potential immunotherapeutic strategy for the treatment of cancer, as Shp1 inhibition would be predicted to unleash both innate and adaptive immunity against tumor cells. Antibodies blocking the interaction between CD47 on tumor cells and SIRPα on macrophages enhance macrophage phagocytosis, show efficacy in preclinical tumor models, and are being evaluated in the clinic. Here we found that Shp1 bound to phosphorylated peptide sequences derived from SIRPα and transduced the anti-phagocytic signal, as Shp1 loss in mouse bone marrow-derived macrophages increased phagocytosis of tumor cells *in vitro*. We also generated a novel mouse model to evaluate the impact of global, inducible *Ptpn6* deletion on anti-tumor immunity. We found that inducible Shp1 loss drove an inflammatory disease in mice that was phenotypically similar to that seen when *Ptpn6* is knocked out from birth. This indicates that acute perturbation of Shp1 *in vivo* could drive hyperactivation of immune cells, which could be therapeutically beneficial, though at the risk of potential toxicity. In this model, we found that Shp1 loss led to robust anti-tumor immunity against two immune-rich syngeneic tumor models that are moderately inflamed though not responsive to checkpoint inhibitors, MC38 and E0771. Shp1 loss did not promote anti-tumor activity in the non-inflamed B16F10 model. The observed activity in MC38 and E0771 tumors was likely due to effects of both innate and adaptive immune cells. Following Shp1 deletion, we observed increases in intratumoral myeloid cells in both models, which was more striking in E0771 tumors. E0771 tumors also contained an increased ratio of effector to regulatory T cells following Shp1 loss. This was not observed for MC38 tumors, though we did find increased levels of IFNγ, a cytokine produced by effector T cells, in these tumors. Overall, our preclinical data suggested that targeting Shp1 may be an attractive therapeutic strategy for boosting the immune response to cancer via a mechanism involving both innate and adaptive leukocytes.

## Introduction

The tumor immune microenvironment is a complex milieu comprised of both innate and adaptive immune cells. Activation of innate or adaptive immune cells can enhance an anti-tumor response. For example, enhancing the ability of effector T lymphocytes to kill tumor cells using checkpoint inhibitors has achieved success in the clinic (1). Therapies that enhance macrophage effector function, such as by targeting the “don't eat me” molecule CD47 on tumor cells to enhance macrophage phagocytosis, are being evaluated in clinical trials and are showing signs of activity in hematological malignancies in combination with opsonizing antibodies (2–5). Promotion of macrophage phagocytosis is a major mechanism of action of many antibodies in cancer therapies (6). The simultaneous targeting of both innate and adaptive immune cells to increase antitumor immunity represents as an exciting and promising therapeutic strategy for cancer.

The protein tyrosine phosphatase Src homology region 2 domain-containing phosphatase-1 (Shp1) is a potential immunotherapeutic target (7, 8). Shp1 is broadly expressed in the hematopoietic compartment and acts as a negative regulator of signaling in both innate and adaptive immune cells (9, 10). Thus, alterations in Shp1 have the potential to impact an anti-tumor immune response in several different ways. Shp1 exerts its inhibitory signaling function by binding to phosphorylated immunoreceptor tyrosine-based inhibitory motifs (ITIMs) on a variety of immunoreceptors. Upon binding and activation, Shp1 dephosphorylates its substrates, thereby transducing inhibitory signals that restrict immune cell function (9, 10). Consistent with this, mouse models with spontaneous mutations in the gene encoding Shp1, *Ptpn6*, that affect its expression or function develop an inflammatory/autoimmune disease associated with hyperactivation of multiple types of immune cells. The *motheaten* (*Ptpn6*^me/me^) mouse was the first *Ptpn6*-mutant mouse model described, and the *me* mutation results in loss of Shp1 protein (11). *Motheaten* mice are runted and die within a few weeks of life from lethal pneumonitis, and the animals also present with a number of other disease features that reflect dysregulation of both innate and adaptive immune cells, such as myelopoiesis, splenomegaly, skin inflammation, and anti-nuclear antibody production (9, 11). Mice with other spontaneous mutations of *Ptpn6*, such as *motheaten-viable* (*Ptpn6*^me−v/me−v^), *Ptpn6*^spin/spin^ and *Ptpn6*^meB2/meB2^ give rise to similar, yet milder inflammatory disease phenotypes (12–14). This has led to the hypothesis that loss of Shp1 activity would promote immune cell activation and enhance effector function in the tumor microenvironment. However, this question has been challenging to address with existing genetic mouse models, as the short lifespan and early-onset disease in *motheaten* and *motheaten-viable* mice would be incompatible with the kinetics of a tumor challenge study. Additionally, there is no selective Shp1 inhibitor available with properties that would enable the pharmacological assessment of Shp1 loss of function on tumor growth. Small molecule Shp1 inhibitors, including TPI-1 and SSG, have been reported (8, 15), but the selectivity and specificity of these inhibitors has not been fully established. Both molecules exhibit relatively low potency and have characteristics consistent with promiscuous Pan-Assay Interference Compounds (PAINS) (16). Specifically, the quinone moiety in TPI-1 and the metal (antimony) in SSG are both capable of non-specific reactivity with cysteine residues, which may account for their apparent inhibitory activity on the cysteine active site of Shp1, but also likely impact many other cellular targets. A recent evaluation of inhibitors of the related receptor tyrosine phosphatase Shp2 using cells that lack Shp2 protein revealed off-target effects (17). Until similar investigations are completed for Shp1 inhibitors, we believe cellular and *in vivo* experiments with these compounds should be interpreted with caution.

The complex *motheaten* phenotype does not arise from loss of Shp1 in any single immune cell subset, as deletion of *Ptpn6* in distinct cell lineages, achieved by crossing a floxed *Ptpn6* mouse to cell type-specific Cre driver lines, does not fully recapitulate the *motheaten* disease features (18–26). However, loss of Shp1 in myeloid cells is required to drive inflammation (9, 18, 27). Shp1 has been proposed to transduce anti-phagocytic “don't eat me” signals downstream of the signal regulatory protein alpha (SIRPα), which is expressed on dendritic cells (DCs) and macrophages, the primary phagocytic cells of the immune system (28, 29). Upon recognition of its ligand CD47, the ITIMs of SIRPα become phosphorylated. This allows for recruitment of Shp1 and activation of its phosphatase activity, leading to downregulation of signals from phagocytic receptors such as Fc receptors, thereby inhibiting phagocytosis (30, 31). Consistent with this, it has been shown that alveolar macrophages from *me* mice exhibit increased phagocytosis of apoptotic cells (32), suggesting that Shp1 loss enhances phagocytic activity. Whether Shp1-deficient macrophages from other anatomical sites also exhibit increased phagocytosis has yet to be determined. Furthermore, it is unknown whether Shp1 loss can augment phagocytosis to a similar degree as antibody blockade of the CD47-SIRPα interaction, or even have an additive effect in combination with pro-phagocytic signaling that is stimulated by the Fc portion of the blocking antibodies binding to Fc receptors on phagocytes. We aimed to address these questions herein and found that Shp1 could bind to phosphorylated peptide sequences derived from SIRPα in a manner that activated its phosphatase activity, and that Shp1-deficient macrophages exhibited enhanced phagocytosis in a manner comparable to that of CD47-SIRPα blockade. There is strong preclinical evidence that blocking the CD47-SIRPα interaction with an antibody enhances phagocytosis and restricts the growth of tumors *in vivo* (5, 33, 34) but whether Shp1 loss in tumor-infiltrating immune cells would similarly enhance anti-tumor immunity remains an open question.

Here we report on the generation of a novel mouse model that facilitated global, inducible deletion of *Ptpn6* in adult mice, and we used this model to uncover a role for Shp1 in anti-tumor immunity. We found that a *motheaten*-like disease consistent with hyperactivation of immune cells occurred when *Ptpn6* deletion was induced in adult mice. Lastly, we report that inducible deletion of *Ptpn6* drove anti-tumor immunity against several syngeneic tumor cell lines, with corresponding alterations in the frequency and/or activity of both myeloid and T lymphocytes in the tumor immune microenvironment. Overall, our data suggest that Shp1 restricts immune cell activity in the tumor microenvironment, and that pharmacological inhibition of Shp1 could lead to activation of both innate and adaptive immune cells to promote anti-tumor immunity.

## Materials and Methods

### Mice

*Ptpn6*^fl/fl^, Cx3cr1-Cre, and Rosa26^LSL−YFP^ mice have been described elsewhere (25, 35, 36). Rosa26-CreERT2 and dLck-Cre mice were obtained from the Jackson Laboratory (37, 38). C57BL/6 mice used for controls were obtained from Jackson Laboratories or Envigo. Mice were kept in specific pathogen-free facilities at the University of California, San Francisco (UCSF), Revolution Medicines or Taconic Biosciences, and cared for in accordance with institutional guidelines. All genotypes of mouse strains used were confirmed by PCR analysis of tail DNA.

### Cell Lines

DLD1, Raji, THP-1 and B16F10 cells were obtained from the ATCC. DLD1 and Raji cells were cultured in RPMI with 10% FCS and 1% penicillin/streptomycin. THP-1 cells were cultured in RPMI with 10% FCS, 1% penicillin/streptomycin and 100 μg/ml Normocin. B16F10 cells were cultured in DMEM with GlutaMAX™ (Gibco) containing 10% FCS (Gibco). MC38 cells were obtained from Kerafast and were used in all studies except those in Supplementary Figure 7, which employed cells that were a gift from Jim Allison (UT MD Anderson Cancer Center), and grown in DMEM with GlutaMAX™ (Gibco) containing 10% FCS (Gibco) and penicillin/streptomycin. E0771 cells were obtained from CH3 Biosystems and cultured in RPMI 1640 with GlutaMAX™ (Gibco) containing 10% FCS (Gibco) and 20 mM HEPES. All cell lines were confirmed to be negative for Mycoplasma species by PCR (IDEXX Bioanalytics) and kept in a humidified incubator with 5% CO_2_ at 37°C.

### Biochemical Phosphatase Assay

Full length wild type SHP1 and all mutants were expressed with C-terminal 6-His tags in E. coli and purified by nickel affinity chromatography using standard techniques (ATUM, Newark, CA). The SIRPα di-phosphopeptide corresponding to residues 427 to 460 of human SIRPα (H_2_N- ITpYADLNLP-PEG8-HTEpYASIQTSK-NH_2_) was synthesized by ThermoFisher Custom Peptides (Carlsbad, CA). The catalytic activity of SHP1 was monitored using the fluorogenic small molecule substrate DiFMUP (ThermoFisher) in 96-well, black polystyrene plates (Corning). The assay was performed in 55 mM HEPES pH 7.2, 100 mM NaCl, 0.5 mM EDTA, 1 mM DTT, 0.001% Brij35, 0.002% BSA, 0.1% DMSO, 20 μM DiFMUP, 0.039 to 5.0 nM enzyme, and 0 to 3,000 nM SIRPα peptide, mixed immediately prior to reading the plate in kinetic mode on a SpectraMax M5 plate reader (Molecular Devices) for 6 min using excitation and emission wavelengths of 340 nm and 450 nm. Plots of initial velocity vs. [SHP1] were fit using linear regression to determine specific activity. Plots of specific activity vs. [SIRPα peptide] were fit using a 4-parameter concentration-response model in GraphPad Prism 8.42, with the upper baseline constrained to the specific activity of the fully activated SHP1 mutant E74K.

### Generation and Polarization of Mouse Bone Marrow-Derived Macrophages

Bone marrow was flushed from mouse femurs and tibias with Ca^2+^ and Mg^2+^-free Hepes-buffered saline solution (HBSS). Pellets were stored at −80°C for protein analysis by western blot, or were frozen in liquid nitrogen in 10% DMSO/90% FCS for cell culture experiments. For *in vitro* differentiation of bone marrow-derived macrophages, the method was adapted from (39). Cells were plated at 2 × 10^6^ cells per 10 cm dish in 10 ml macrophage media consisting of αMEM (Gibco) supplemented with 10% FCS, 1% penicillin/streptomycin, 2 mM L-glutamine and 10 ng/ml M-CSF (Peprotech). Functional assays were performed starting on day 7 of culture. For polarization, macrophages were removed from plates using Cell Dissociation Buffer (Gibco), washed, and plated in either M0 conditions (macrophage media), M1-polarizing conditions (macrophage media supplemented with 100 ng/ml LPS and 20 ng/ml recombinant IFNγ) or M2-polarizing conditions (macrophage media supplemented with 20 ng/ml recombinant murine IL-4) for 24 h before analysis. To measure CD206 expression on M2 polarized mouse macrophages, cells were removed from plates using Cell Dissociation Buffer (Gibco) and stained for subsequent analysis by flow cytometry as outlined below. To measure cytokine production by M1 polarized macrophages, supernatants were harvested and analyzed by multiplexed ELISA.

### Generation and Polarization of Human Monocyte-Derived Macrophages

Human monocytes were isolated from peripheral blood mononuclear cells using Miltenyi Biotech Classical Monocyte Isolation kit #130-117-337 or Miltenyi Biotec CD14 MicroBeads #130-050-201. Monocytes were cultured in RPMI (Sigma) supplemented with 10% heat-inactivated FCS (Gibco), 50 μM β-mercaptoethanol (Gibco) and 50 ng/ml M-CSF (R&D Systems). Cells were cultured for 5–6 days in 6-well plates (Costar). For siRNA-mediated knockdown of *PTPN6*, cells were washed and resuspended in antibiotic-free macrophage medium. siRNA oligos were obtained from Horizon Discovery (SMARTpool ON-TARGETplus human PTPN6 #L-009778-00-0020 and ON-TARGETplus non-targeting pool #D-001810-10-20). siRNAs and the transfection reagent RNAi Max (Invitrogen) were added to Opti-MEM medium (Gibco) and incubated for 15 min before drop-wise addition to cells. Cells were incubated with transfection mixture for 6 h at 37°C and were analyzed on day 6. For macrophage polarization, macrophage media was supplemented with the following factors 2 days after siRNA transfection: for M1, 20 ng/ml recombinant human IFNγ (R&D Systems) and 100 ng/ml LPS (Sigma) and for M2, 20 ng/ml recombinant human IL-4 (R&D Systems) and 10 ng/ml recombinant human IL-13 (R&D Systems). Supernatants were harvested from polarized macrophages and secreted cytokines were measured by Luminex Multiplex Immunoassay (Bio-Rad) with reagent kits for IL-12p70 (171B5011M), TNFα (171B5026M), IL-6 (171B5006M), and IL-10 (171B5010M). CD206 expression on M2 polarized human macrophages was determined by fixation with 4% formaldehyde and staining with anti-CD206 (Abcam #Ab64693), followed by Alexa Fluor 647-conjugated secondary antibody. Cells were co-stained with Hoescht 33342 staining solution and Alexa Fluor 488-Phalloidin (Thermofisher). Fluorescent imaging was performed with the IN Cell Analyzer 2200 (GE Healthcare) and average fluorescence intensity was quantified.

### Phagocytosis Assay

For assays with murine bone marrow-derived macrophages, cells were harvested from tissue culture dishes using Cell Dissociation Buffer (Gibco) or Accutase (Corning) and resuspended in RPMI. Target cells were cultured as previously described and labeled with 25 nM CellTrace Far Red proliferation dye (ThermoFisher) for 20 min at 37°C, at a density of 1 × 10^6^ cells/ml. Dye was quenched and cells were resuspended in RPMI. Target cells were pre-incubated with opsonizing antibodies (anti-CD47 clone B6H12, eBioscience; mouse IgG1k isotype control, clone P3.6.2.8.1, Thermo Fisher; anti-CD20, Selleckchem), and macrophages were pre-incubated with Fc block (anti-CD16/32, BD Biosciences, clone 2.4G2) as indicated. Macrophages and labeled target cells were co-cultured for 4 h at 37°C in low-adherence 96-well plates at a ratio of 1:2, respectively. Cells were then washed and stained with 4′,6′-Diamidino-2-Phenylindole, Dihydrochloride (DAPI) prior to analysis by flow cytometry (Cytoflex, Beckman Coulter).

For phagocytosis assays with human macrophages treated with siRNA, macrophages were harvested by scraping and incubated with target cells that were pre-labeled with CellTrace Violet proliferation dye (ThermoFisher) for 4 h at 37°C. Cells were stained with LIVE/DEAD™ Fixable Far Red (Invitrogen) and anti-human CD11b (Biolegend, clone ICRF44) prior to analysis by flow cytometry. Phagocytic index was determined by gating on live, single cells, and identifying CellTrace Far Red^+^ YFP^+^ (mouse) or CellTrace Violet^+^ CD11b^+^ (human) macrophages.

### Generation and Analysis of *PTPN6*-Deficient THP-1 Cells

*PTPN6*-deficient THP-1 cell pools were generated by transfection of Cas9RNPs targeting the *PTPN6* locus. Cas9RNP production and nucleofection was performed as previously described (40). The *PTPN6* locus was targeted using the following sgRNA sequence: TCACGCACAAGAAACGTCCA and a non-targeting (NT) sgRNA was used to generate a control cell line. Single cell clones were selected by limiting dilution and SHP1 loss was verified in monoclonal THP-1 cell lines by Western blot analysis.

To measure cytokine production, control and *PTPN6-*deficient THP-1 cell lines were plated at a density of 5 × 10^4^ cells per well in a 96-well plate and rested at 37°C for 1 h before adding 1 μg/ml LPS (from E. coli 0111:B4, Invivogen). Cells were incubated at 37°C for 20 h, after which supernatant was removed and frozen at −80°C. Supernatants were thawed and assayed for TNF-α, IL-1β, IL-6, and IFNγ using a V-Plex Human ProInflammatory Panel I Kit on the Meso Scale Discovery (MSD) platform.

### Western Blot Analysis

Tissue lysates were prepared from tissues snap-frozen in liquid nitrogen and powdered using a mortar and pestle. Powdered tissue was lysed in NP40 buffer supplemented with protease/phosphatase inhibitor cocktail (ThermoFisher, #78446) for 30 min on ice, then centrifuged at 4°C for 15 min at 21,000 × g to remove insoluble material. For peripheral blood lysates, blood was collected in K2-EDTA tubes (BD Biosciences), washed with PBS, and centrifuged for 5 min at 400 × g. Red blood cells in the pellet were lysed using RBC lysis solution (Miltenyi Biotec) following the manufacturer's protocol, or using ACK lysis buffer (150 mM NH_4_Cl, 10 mM KHCO_3_, 0.1 mM EDTA pH 7.2–7.4), and centrifuged for 5 min at 400 × g. The leukocyte-containing pellet was lysed in NP40 buffer as detailed above or lysed directly in Laemmli sample buffer. Total protein concentration of cell lysates was determined using the Pierce BCA Protein Assay kit (ThermoFisher). Equal amounts of protein were loaded onto 4–12% SDS-PAGE gradient gels for separation, transferred to nitrocellulose or PVDF membranes, blocked, and immunoblotting was performed using the following primary antibodies: anti-SHP1 (Abcam, clone Y476 #ab325599), anti-Shp1 (Santa Cruz, C19 #sc-287), anti-Shp1 (LSBio, #LS-C358839), anti-Erk1/2 (Cell Signaling, clone L34F12 #4696), or anti-Erk2 (Santa Cruz, D2 #sc1647) at 4°C overnight. Blots were probed with secondary antibodies: goat anti-rabbit IgG (LI-COR, 800CW) and goat anti-mouse IgG (LI-COR, 680RD), washed and scanned using the LI-COR Odyssey CLx. Protein bands were quantified using LI-COR Image Studio Acquisition Software.

### Inducible Mouse Model of *Ptpn6* Deletion

For maximum Cre-mediated loss of Shp1, *Ptpn6*^fl/fl^, and *Ptpn6*^fl/fl^ ERT2-cre mice were treated with tamoxifen (Toronto Research Chemicals) at 200 mg/kg bid for 4 days by oral gavage. Tamoxifen was dissolved in corn oil (Sigma) at 20 mg/ml by incubation at 37°C for 8–12 h. Mouse bodyweight was monitored every 2–3 days.

### PrimeFlow Analysis

Probes for murine *Ptpn6* (AF647 conjugate, Assay ID: VB1-3030134-PF) and murine beta-actin (AF750 conjugate, Assay ID: VB6- 12823-PF) were from ThermoFisher Scientific. Single cell suspensions of splenocytes were prepared and 2 × 10^6^ cells were plated per well of a 96-well V-bottom plate. Cells were stained with LIVE/DEAD™ Fixable Aqua (ThermoFisher), blocked with anti-CD16/32 (BD Biosciences) and stained with the following antibodies to surface markers: Panel 1: NKp46 FITC, TCRβ PE-Cy7, CD25 PE, B220 BV421, CD8 BV650, CD4 BV711 or Panel 2: F4/80 FITC, Ly6g PE-Cy7, CD11c PE, CD11b e450, MHCII BV711 in Superbright staining buffer (ThermoFisher). Cells were fixed and permeabilized using PrimeFlow Assay Kit buffers following the manufacturer's instructions (ThermoFisher). Target probes were hybridized for 2 h at 40°C then cells were washed and stored overnight at 4°C in PrimeFlow RNA Wash Buffer with RNase Inhibitor 1. The following day, signal amplification steps were performed according to manufacturer's protocol and cells were analyzed by flow cytometry on a BD Fortessa flow cytometer. Data was analyzed using FlowJo (Treestar) and the mean fluorescence intensity (MFI) of the *Ptpn6* mRNA probe was determined in the following cell types: PMNs (CD11b^+^ Ly6g^+^), Tregs (B220^−^ TCRβ^+^ CD4^+^ CD25^+^ FoxP3^+^), CD4^+^ T cells (B220^−^ TCRβ^+^ CD4^+^ CD8^−^), and CD8^+^ T cells (B220^−^ TCRβ^+^ CD4^−^ CD8^+^).

### Flow Cytometry Analysis

For confirmation of CD47 expression on target cell lines, DLD1 and Raji cells were stained with anti-human CD47 clone B6H12 or mouse IgG1_k_ isotype control. MC38 cells were stained with anti-mouse CD47 clone miap301 or rat IgG2a_k_ isotype control.

To assess CD206 staining on polarized mouse macrophages, cells were detached using Cell Dissociation Buffer as previously described. Cells were stained with a fixable viability dye (ThermoFisher) according to the manufacturer's protocol, blocked with anti-mouse IgG and anti-CD16/32 (BD Biosciences, clone 2.4G2,) and stained with antibodies to surface markers. Cells were then fixed in FoxP3 fixation buffer (eBiosciences) and stained with anti-CD206-PE (Biolegend, clone C068C2). Cells were analyzed using a Cytoflex flow cytometer (Beckman Coulter).

Single cell suspensions of splenocytes were prepared by homogenizing spleens between two frosted microscope slides, followed by passage through a 70 μM cell strainer. Bronchoalveolar lavage (BAL) cells were obtained with five 1 ml flushes of the lungs with ice-cold 5 mM EDTA in PBS. Red blood cells were removed from cell suspensions by lysis with ACK buffer (150 mM NH_4_Cl, 10 mM KHCO_3_, 0.1 mM EDTA pH 7.2–7.4). Cells were resuspended in HBSS containing 2% (vol/vol) FCS, 20 mM HEPES and 1 mM EDTA and maintained at 4°C. Cell numbers were determined by using a Nucleocounter™ (Chemometec). Tumors were disaggregated using the GentleMACS Mouse Tumor Dissociation kit (Miltenyi Biotech) and resuspended in HBSS containing 2% FCS, 20 mM HEPES and 1 mM EDTA. For surface staining, cells were pre-incubated with 0.5 μg anti-CD16/32 antibody (clone 2.4G2, UCSF Hybridoma core) and 100 μg mouse IgG (Sigma) for 15 min to block non-specific binding, followed by addition of the following fluorescently-conjugated antibodies: From BD Biosciences; anti-mouse γδTCR FITC (Clone GL3, Cat# 553177), anti-mouse CD11c PE (Clone HL3, Cat# 553802), anti-mouse NK1.1 PE-Cy7 (Clone PK136, Cat# 553165), anti-mouse CD45R (B220) AF700 (Clone RA3-6B2 Cat# 557957), anti-mouse Ly6c AF780 (Clone AL-21 Cat# 560596), anti-mouse CD8 BV650 (Clone 53-6.7 Cat# 563234), anti-mouse I-A/I-E BV711 (Clone M5/114 Cat# 563414), anti-mouse TCRβ BV711 (H57-597 Cat# 563135), anti-mouse CD4 BV786 (Clone GK1.5 Cat# 563331), anti-mouse CD11b BUV395 (Clone M1/70 Cat# 563553). From Thermo Fisher; anti-mouse TCRβ PerCP-Cy5.5 (Clone H57-597, Cat# 45-5961), anti-mouse CD45R (B220) PerCP-Cy5.5 (Clone RA3-6B2, Cat# 45-0452), anti-mouse FoxP3 PE (Clone FJK-16S, Cat# 12-5773), anti-mouse CD25 APC (Clone PC61.5 Cat# 17-0251), anti-mouse CD3 AF780 (Clone 145-2C11 Cat# 47-0031), anti-mouse CD44 eFluor450 (Clone IM7 Cat# 48-0441). From Bio-Rad; anti-mouse F4/80 AF647 (Clone CI:A3-1, Cat# MCA497A647). From Biolegend; anti-mouse Ly6g Pacific Blue (Clone 1A8 Cat# 127612), anti-mouse CD45 BV605 (Clone 30-F11 Cat# 103139). Viability was assessed by staining with Live/Dead fixable Aqua (Thermo Fisher) or DAPI exclusion. Cells were fixed and permeabilized for intracellular staining using the FoxP3/Transcription Factor staining kit (Thermo Fisher). AccuCheck Counting Beads (Thermo Fisher) were added to samples prior to acquisition to obtain cell counts in some cases. All flow cytometry of material from the inducible mouse model was performed on a BD Fortessa flow cytometer. Analysis of flow cytometry data was done using Flowjo (Treestar). Tumor immune cell populations were identified by the gating strategy shown in Supplementary Figure 8.

### Histology and Immunohistochemistry

Mouse lung lobes were inflated with formalin and fixed for 48 h prior to paraffin embedding, sectioning and staining with H&E. Images were collected using a Zeiss Axio Imager M2 and Zen Pro software. For staining with anti-F4/80 (Cell Signaling, Cat. #70076), lung lobes were fixed with formalin for 24 h prior to paraffin embedding and sectioning. Citrate-based pH 6.2 Heat-Induced Epitope Retrieval was used on the Biocare *intelliPATH* automated staining platform using the manufacturer's recommended settings. The sections were incubated with Biocare Peroxidase Blocker (Biocare, Cat. #PX968) and Background Punisher (Biocare, Cat. #BP974M) to block non-specific background staining. For the detection of rabbit primary antibodies, MACH4 HRP-polymer Detection System (Biocare, Cat. #MRH534) was used. The chromogenic detection and counterstaining kits IntelliPATH FLX DAB chromogen (Biocare, Cat. #IPK5010) and IntelliPATH Hematoxylin (Biocare Medical, Cat. #XMF963) were used.

Tumors were dissected from mice, bisected lengthwise, and formalin fixed for 24 h prior to paraffin embedding, sectioning and staining with H&E, or anti-mouse CD8 (Cell Signaling, Cat. #98941) as detailed above. Stained slides were digitized with a TissueScope LE whole slide scanner (Huron Digital Pathology) and images captured with Huron Viewer software. Quantification of CD8 staining was performed with the HALO® Image Analysis software from Indica labs using the CytoNuclear module.

### Growth of Syngeneic Tumor Lines *in vivo*

Cells for *in vivo* use were maintained under limited passage (<5) from original stocks.

5 × 10^4^ B16F10 cells in PBS were mixed 1:1 with ice cold Matrigel (Corning) and 100μl was injected subcutaneously into the inguinal area. 1 × 10^6^ E0771 cells in 100μl PBS were injected into the mammary fat pad. 5 × 10^5^ MC38 cells in 200μl HBSS were injected subcutaneously into the inguinal area or flank except for Supplementary Figure 7 where 5 × 10^5^ MC38 cells in 100μl 1:1 Matrigel:PBS were injected. Tumor volume was measured with digital calipers using the formulas (width^2^ × length) for Figures 4B,E and Supplementary Figures 6A,7B, or (width^2^ × length/2) for Figure 4H and Supplementary Figures 6B,C. Mice were sacrificed when tumor volume >2,000 mm^3^ or body weight dropped below 80% of initial weight.

### Antibody Treatment *in vivo*

For studies with MC38 tumors, wild type C57BL/6 mice were implanted with MC38 cells as described above. When tumor volume reached an average of 75 mm^3^, mice were randomized into groups of *n* = 15 mice. For studies with E0771 tumors, *Ptpn6*^fl/fl^ mice were implanted as described above. For anti-PD1 studies: mice were dosed intraperitoneally (IP) with anti-PD1 (clone RMP1-14, BioXCell) or rat IgG2a isotype control (clone 2A3, BioXCell) at 10 mg/kg twice weekly (BIW). For anti-CD47 studies, mice were dosed IP with anti-CD47 (clone miap301, BioXCell) or rat IgG2a isotype control (clone 2A3, BioXCell) at 20 mg/kg BIW for 2 weeks as described in Liu et al. (41).

### Analysis of Cytokine Levels in Murine Tumor Lysates

Snap-frozen tumors (0.1 g) were homogenized in 1 ml of buffer containing protease inhibitors using a Precellys homogenizer. Total protein concentration of the homogenates was determined using a BCA assay. Equal volumes of the homogenates were immediately assessed for cytokine/chemokine protein expression using a multiplex bead assay (Luminex, MILLIPLEX MAP Mouse Cytokine/Chemokine Magnetic Bead Panel). Results were normalized to the total concentration of protein as determined by BCA assay.

### Statistical Analysis

Statistical analysis was performed using GraphPad Prism (GraphPad Software). Data were analyzed by unpaired *t*-test. For multiple group comparisons, an Analysis of Variance (ANOVA) was performed. The *p*-values for ANOVA analysis were calculated using Dunnett's test. Data were presented as mean ± SEM unless stated otherwise. Statistical significance was indicated as ^*^p < 0.05, ^**^p < 0.01, ^***^p < 0.001, and ^****^p < 0.0001.

## Results

### Shp1 Phosphatase Transduces the “Don't Eat Me” Signal Downstream of SIRPα

**Figure 1:**
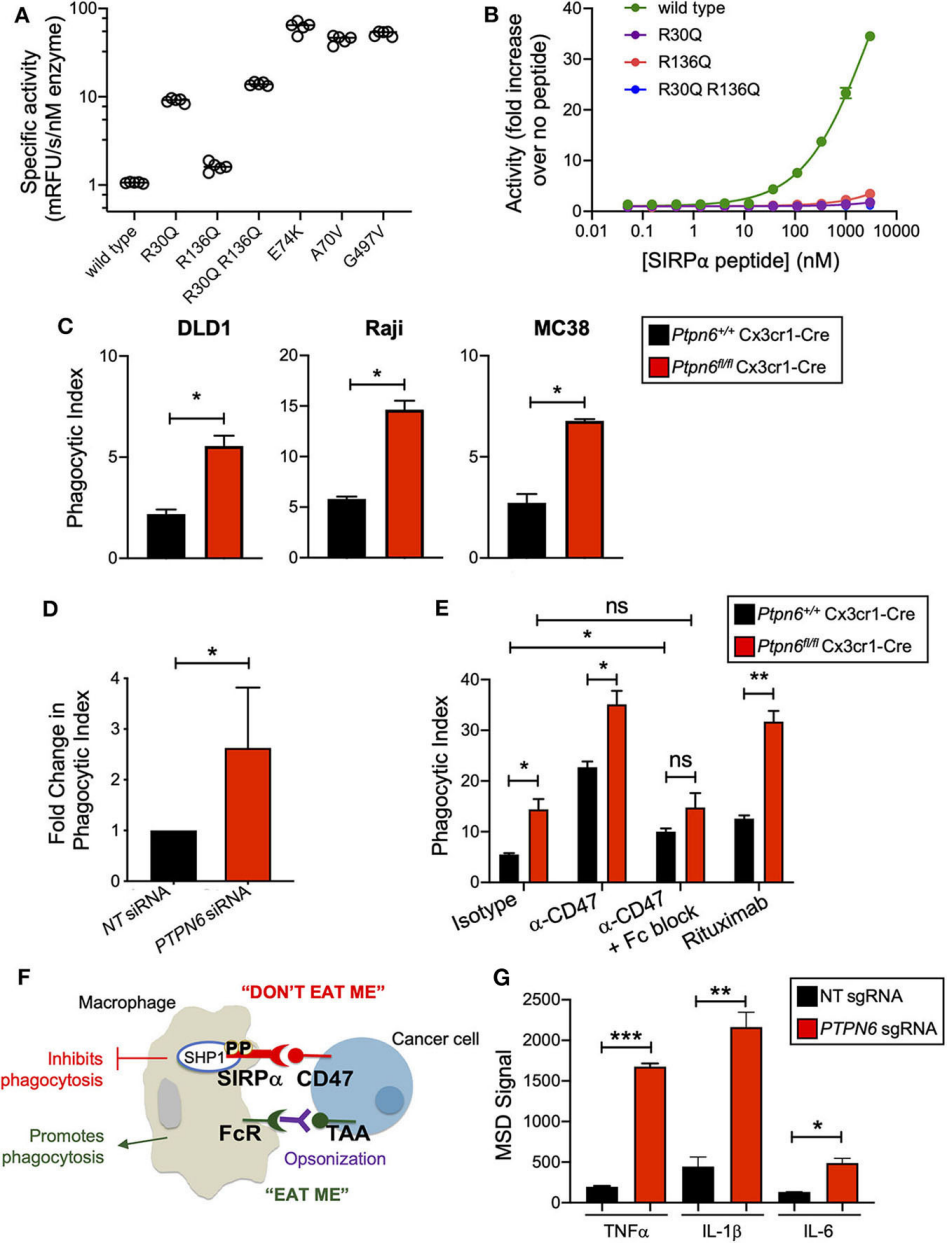
Shp1 phosphatase transduces the “don't eat me” signal downstream of SIRPα. **(A)** Specific activity on the fluorogenic small molecule DiFMUP of purified, full-length, wild-type SHP1, phosphotyrosine binding-deficient mutants (R30Q, R136Q, and R30Q R136Q), and substitutions homologous to known activating mutations in SHP2 (E74K, A70V, and G497V) in the absence of activating concentrations of SIRPα peptide. **(B)** Increase in biochemical activity of SHP1 upon titration with a synthetic peptide corresponding to residues 427–460 of SIRPα, with tyrosines 429 and 453 phosphorylated. Shown are wild type SHP1 (green) and mutants that eliminate phosphotyrosine binding to the N-SH2 (R30Q, purple) and C-SH2 (R136Q, red) domains, and the double mutant (R30Q R136Q, blue). Lines represent fit to a 4-parameter concentration response model. **(C)** Flow cytometry-based *in vitro* phagocytosis assay. Murine bone marrow-derived macrophages (BMDMs) were co-cultured with DLD1, Raji, or MC38 target cells for 4 h prior to analysis by flow cytometry. Phagocytic index is defined as the number of macrophages that had taken up target cells divided by the total number of macrophages. Data is representative of 5 independent experiments with 2 mice/group. **(D)** as in **(C)** but with human peripheral blood monocyte-derived macrophages with *PTPN6* knockdown by siRNA, compared to non-targeting siRNA control. DLD1 were used as target cells. Data is normalized and combined from 4 independent experiments with 1–2 donors per experiment. **(E)** As in **(C)**, but target cells were pre-incubated with antibodies (isotype control, anti-CD47, rituximab, where indicated) and murine BMDMs were pre-incubated with Fc block (where indicated) prior to coculture. Raji were used as target cells. Data is representative of two to five independent experiments with 2 mice/group. **(F)** Cartoon schematic of the balance of signals that impact macrophage phagocytosis. Interaction of SIRPα on the macrophage with CD47 on the cancer target cell leads to an anti-phagocytic “don't eat me” signal that is transduced by SHP1. Macrophages can also receive pro-phagocytic “eat me” signals via stimulation of certain Fc receptors. This can occur when a tumor-associated antigen (TAA) is bound by an antibody, opsonizing the tumor target cell. The Fc portion of the opsonizing antibody can bind to macrophage Fc receptors, driving the “eat me” signal. **(G)** Immunoassay (MSD) for cytokine secretion by wild type (non-targeting, NT, sgRNA) and SHP1 knockout (*PTPN6* sgRNA) THP1 cells. Cells were stimulated with 1μg/ml LPS for 20 h prior to harvesting of culture supernatants. Data is representative of four independent experiments with 3 technical replicates per condition per experiment. Error bars represent SEM, *p < 0.05, **p < 0.01, ***p < 0.001.

Shp1 has a similar domain arrangement and tertiary structure to its paralog Shp2, which is known to adopt an autoinhibited conformation that is activated by binding of both SH2 domains to bis-phosphorylated, tyrosine containing sequence motifs (e.g., ITIMs and ITAMs) (30, 31). We investigated whether the activity of human SHP1 protein was similarly regulated by binding to phosphorylated motifs of interacting proteins such as SIRPα. First, we determined whether SHP1 adopted an autoinhibited conformation in the absence of SH2 domain binding. Multiple activating mutants of SHP2 occur in the inherited disorder Noonan Syndrome and sporadically in cancer genomes, including A72V, E76K, and G503V. Many of these mutations activate SHP2 by destabilizing the autoinhibited conformation (42). We made the same substitutions at homologous positions in SHP1 (A70V, E74K, and G497V). Compared to wild type SHP1, all three variants exhibited >40-fold increased activity on the fluorogenic synthetic substrate 6,8-Difluoro-4-Methylumbelliferyl Phosphate (DiFMUP) (Figure 1A), indicating that these mutations disrupted an autoinhibited conformation of SHP1 similar to that of SHP2. We then investigated whether SHP1 could be activated by binding of phosphotyrosine-containing sequence motifs from SIRPα, using a synthetic peptide comprised of the sequence motifs surrounding phosphorylated tyrosines 429 and 453 of human SIRPα, separated by a flexible PEG8 linker. SHP1 activity on DiFMUP increased in a concentration dependent manner with SIRPα peptide, up to 35-fold at the highest concentration tested (3 μM) (Figure 1B). To investigate the role of specific interactions with the SH2 domains of SHP1 in this activation, we replaced an arginine residue critical for phosphotyrosine binding (43) with glutamine in the N-SH2 domain (SHP1 R30Q), C-SH2 domain (SHP1 R136Q) and both domains (SHP1 R30Q R136Q). All engineered variants had catalytic activity, indicating properly folded protein (Figure 1A). The basal activity of R136Q was similar to wild type, whereas the activity of R30Q and the double mutant was ~10-fold higher than wild type, possibly due to allosteric disruption of the autoinhibited conformation by the N-SH2 domain binding site mutation. Mutations in the SH2 domains greatly attenuated activation by SIRPα peptide binding; activity of SHP1 R136Q increased 3.5-fold, R30Q 1.8-fold, and R30Q R136Q 1.1-fold at 3 μM SIRPα peptide (Figure 1B). Taken together, these observations suggested that SHP1 adopts an autoinhibited conformation similar to SHP2, and that SHP1 phosphatase activity can be induced by binding to phosphorylated SIRPα.

We next wanted to test the hypothesis that Shp1 loss, and thus loss of the anti-phagocytic signal downstream of SIRPα, could enhance macrophage phagocytosis. To obtain *Ptpn6*-deficient macrophages, we crossed *Ptpn6*^fl/fl^ mice to Cx3cr1-Cre Rosa26^LSL−YFP^ and generated YFP^+^ bone marrow-derived macrophages (BMDMs) from these animals as well as from wild type (WT) control mice (*Ptpn6*^+/+^ Cx3cr1-Cre Rosa26^LSL−YFP^). We consistently achieved >85% reduction in Shp1 protein levels in *Ptpn6*-deleted BMDMs (Supplementary Figure 1A). To evaluate phagocytosis, we adapted a flow cytometry-based assay (44) wherein YFP-expressing macrophages were co-cultured with fluorescently-labeled cancer target cells, and the frequency of dual-labeled macrophages in the culture was measured, indicative of target cell uptake and referred to as “phagocytic index” (Supplementary Figure 1B). All target cell lines tested, which included both epithelial and hematopoietic cancer cell lines (DLD1, Raji, MC38) expressed the SIRPα ligand CD47 (Supplementary Figure 1C). *Ptpn6-*deficient BMDMs exhibited a 2–3-fold increase in phagocytic index compared to WT macrophages when co-cultured with DLD1 or Raji human cancer cells as well as the murine syngeneic colon tumor line MC38 (Figure 1C). Similar results were observed with human peripheral blood monocyte-derived macrophages transduced with *PTPN6*-targeted siRNA compared to macrophages transduced with a non-targeting siRNA (Figure 1D). Pre-incubation of murine and human macrophages with anti-CD47 in order to block the “don't eat me” signal led to increased phagocytosis by both WT and *Ptpn6-*deficient macrophages (Figure 1E, Supplementary Figure 1B). Phagocytosis is governed by a combination of anti-phagocytic “don't eat me” signals, such as the one driven by CD47 binding to SIRPα, and by “eat me” signals that come from crosslinking of Fc receptors on macrophages; this triggers a signaling pathway that is pro-phagocytic and leads to engulfment of the target cell [(29); schematized in Figure 1F]. In the experiments shown in Figure 1E, we used an intact anti-CD47 antibody: this can disrupt both the CD47-SIRPα interaction via the variable region of the antibody binding to CD47, and can also bind to macrophage Fc receptors which would enhance phagocytic uptake by triggering the described pro-phagocytic “eat me” signaling pathway. To uncouple the effect of blocking “don't eat me” signals from promoting “eat me” signals, we first pre-incubated macrophages with an antibody that blocks Fc receptors (“Fc block,” α-CD16/32). Here, any increase in phagocytosis would be due to blocking the “don't eat me” CD47-SIRPα signal alone. Consistent with this, pretreating WT macrophages with “Fc block” prior to incubation with target cells and anti-CD47 led to increased phagocytosis compared to cocultures with isotype antibody (Figure 1E). In contrast, *Ptpn6*-deleted macrophages pretreated with Fc block did not drive a further increase in phagocytosis compared to isotype control antibody treatment (Figure 1E). This demonstrated that Shp1 transduced the “don't eat me” signal, and that Shp1 loss-of-function in macrophages drove a similar increase in phagocytosis to that observed with CD47 blockade. Of note, we observed the highest phagocytic index when we combined Shp1 loss with an opsonizing antibody that stimulates an “eat me” signal through macrophage Fc receptors, such as anti-CD47 or anti-CD20 (rituximab) (Figure 1E). This is consistent with the emergent clinical success of combining a non-opsonizing anti-CD47 antibody with rituximab in treating CD20^+^ B cell lymphomas (45).

We also found that SHP1 deletion induced phenotypic changes in macrophages, making them more pro-inflammatory. We knocked out SHP1 in the human monocytic cell line THP-1 using CRISPR-Cas9 and found that clonal SHP1-deficient cells stimulated with lipopolysaccharide (LPS) produced higher levels of the inflammatory cytokines TNFα, IL-1β, and IL-6 compared to THP-1 cells that received a non-targeting (NT) control sgRNA (Figure 1G). We next evaluated the impact of Shp1 loss on BMDMs polarized to the proinflammatory “M1” and alternatively-activated “M2” subtypes by incubation with either LPS and IFNγ, or IL-4, respectively, in addition to the non-polarized “M0” BMDMs used in previous experiments. *Ptpn6*-deficient M0 macrophages expressed lower levels of the mannose receptor CD206 (MRC1), an M2-associated marker that plays a major role in the progression of solid tumors due to immunosuppressive effects and impacts on angiogenesis and metastasis (46, 47). This suggested that *Ptpn6*-deficient macrophages were phenotypically less “M2-like” compared to WT macrophages (Supplementary Figure 1D). We also observed a reduction in CD206 levels on *Ptpn6*-deficient M2 macrophages (Supplementary Figure 1D). Human *PTPN6*-knockdown M2-polarized macrophages also exhibited lower CD206 levels, consistent with our findings in murine macrophages (Supplementary Figure 1E). *PTPN6*-deficient human M1 macrophages trended toward increased secretion of the proinflammatory cytokines IL-6 and TNFα (Supplementary Figure 1F), consistent with published literature (48). We also observed a trend toward increased IL-1β and IL-10 in mouse M1 macrophages, and reduced IL-12p70 (Supplementary Figure 1G), however differences in the experimental methodologies for Shp1 perturbation (siRNA knockdown for human cells and Cre-mediated DNA deletion in the mouse cells) make it hard to draw direct comparisons between our findings in the mouse and human cells. Overall, we found that Shp1 loss in macrophages resulted in a more proinflammatory phenotype and enhanced phagocytic effector function.

### Generation of a Genetically-Engineered Mouse Model for Global, Inducible Deletion of *Ptpn6*

**Figure 2:**
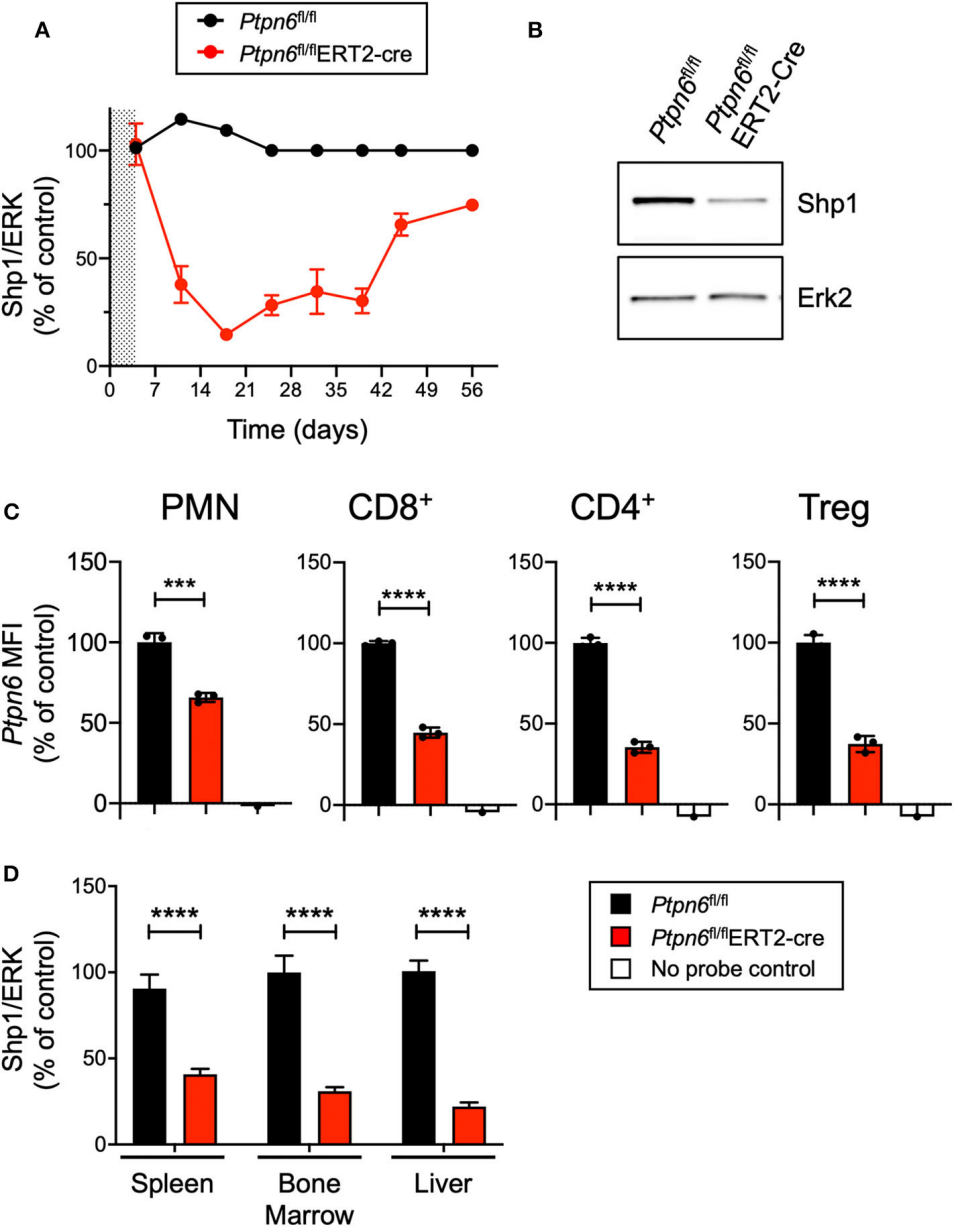
Generation of a genetically-engineered mouse model for global, inducible deletion of *Ptpn6*. **(A)** Shp1 protein relative to total Erk2 protein in peripheral blood cells analyzed over time, from *Ptpn6*^fl/fl^ and *Ptpn6*^fl/fl^ ERT2-Cre mice that had been treated with tamoxifen (200 mg/kg bid for 4 days, represented by shaded area). Data is representative of at least three independent experiments with 4–5 mice pre group. **(B)** Representative immunoblot from A of Shp1 and Erk2 protein levels in whole cell lysates of peripheral blood cells from indicated mice on day 18 following tamoxifen treatment. Lysates were analyzed by SDS-PAGE followed by immunoblotting with indicated antibodies. **(C)** Flow cytometric analysis of *Ptpn6* mRNA levels in the indicated subsets of immune cells from *Ptpn6*^fl/fl^ and *Ptpn6*^fl/fl^ ERT2-Cre mouse spleens on day 12 after MC38 tumor implant (day 19 after initial tamoxifen dose). Data is from one experiment with *n* = 3 mice/group. **(D)** Analysis of Shp1 protein levels by immunoblot in mouse spleen, bone marrow, and liver tissue lysates. Organs were isolated from MC38 tumor-bearing *Ptpn6*^fl/fl^ and *Ptpn6*^fl/fl^ ERT2-Cre mice at study endpoint and tissue lysates were homogenized, then analyzed by SDS-PAGE followed by immunoblotting with indicated antibodies. Quantitated Shp1 protein was normalized to total Erk protein. Data is representative of three independent experiments with 4–10 mice/group. Error bars represent SEM, ***p < 0.001, ****p < 0.0001.

Given that enhancing macrophage phagocytosis has been demonstrated to increase anti-tumor activity in preclinical models (6), we wanted to evaluate the impact of Shp1 loss on tumor growth. *Ptpn6*^me/me^ *or Ptpn6*^mev/mev^ mice have constitutive mutations in Shp1 and could not be used for tumor growth studies because these animals succumb to *motheaten* disease too rapidly to allow for evaluation of tumor growth (9, 11, 14). Thus, to test the effect of Shp1 loss on tumor growth, we generated a model for global, inducible deletion of *Ptpn6*. To achieve this, we crossed the *Ptpn6*^fl/fl^ mice (25) to the *Rosa26*^Cre/ERT2^ strain (37): with this model (referred to as *Ptpn6*^fl/fl^ERT2*-*Cre), the Cre was sequestered in the cytosol by virtue of its fusion with the estrogen receptor (ER), and could only translocate to the nucleus and recombine out the loxP-flanked *Ptpn6* DNA upon administration of the ER ligand tamoxifen (49). We developed a tamoxifen dosing regimen that led to a 70–80% reduction in Shp1 protein levels in peripheral blood cells as measured by immunoblot for Shp1 (Figures 2A,B). This degree of deletion was detectable 2 days following the last dose of tamoxifen treatment and was sustained for 40 days following initial tamoxifen dosing (Figure 2A). Deletion was equivalent in both male and female mice (Supplementary Figure 2). Of note, Shp1 protein levels in the peripheral blood began to increase beyond 40 days post-tamoxifen treatment, approaching wild type levels (Figure 2A). It is possible that *Ptpn6* was not effectively deleted in progenitor cells following tamoxifen treatment, leading to the observed restoration of detectable Shp1 protein. We determined that *Ptpn6* was knocked out in both innate and adaptive immune cells using a flow cytometry assay that enables detection of mRNA as a surrogate for protein expression because there is no available antibody that detects Shp1 protein by flow cytometry. Using mRNA flow cytometry, we could detect reduced *Ptpn6* mRNA levels in several different immune cell subsets, including neutrophils (PMN), CD8^+^ T cells, and both effector and regulatory (Treg) CD4^+^ T cells in the spleens of *Ptpn6*^fl/fl^ERT2-Cre mice 19 days following tamoxifen administration (Figure 2C). The reduction in mRNA levels was concordant with the reduction in protein observed in total peripheral blood cells in Figure 2A. Loss of Shp1 protein was also detected in the peripheral tissues of tamoxifen-treated *Ptpn6*^fl/fl^ERT2*-*Cre mice, including the spleen, bone marrow, and liver (Figure 2D). Overall, we were able to generate a mouse model that allowed for inducible deletion of *Ptpn6* in adult mice and developed an assay to monitor Shp1 protein levels over time in mouse peripheral in blood cells, as well as at study endpoint in peripheral tissues.

### Global, Inducible Deletion of *Ptpn6* Leads to Features of the *Motheaten* Phenotype

**Figure 3:**
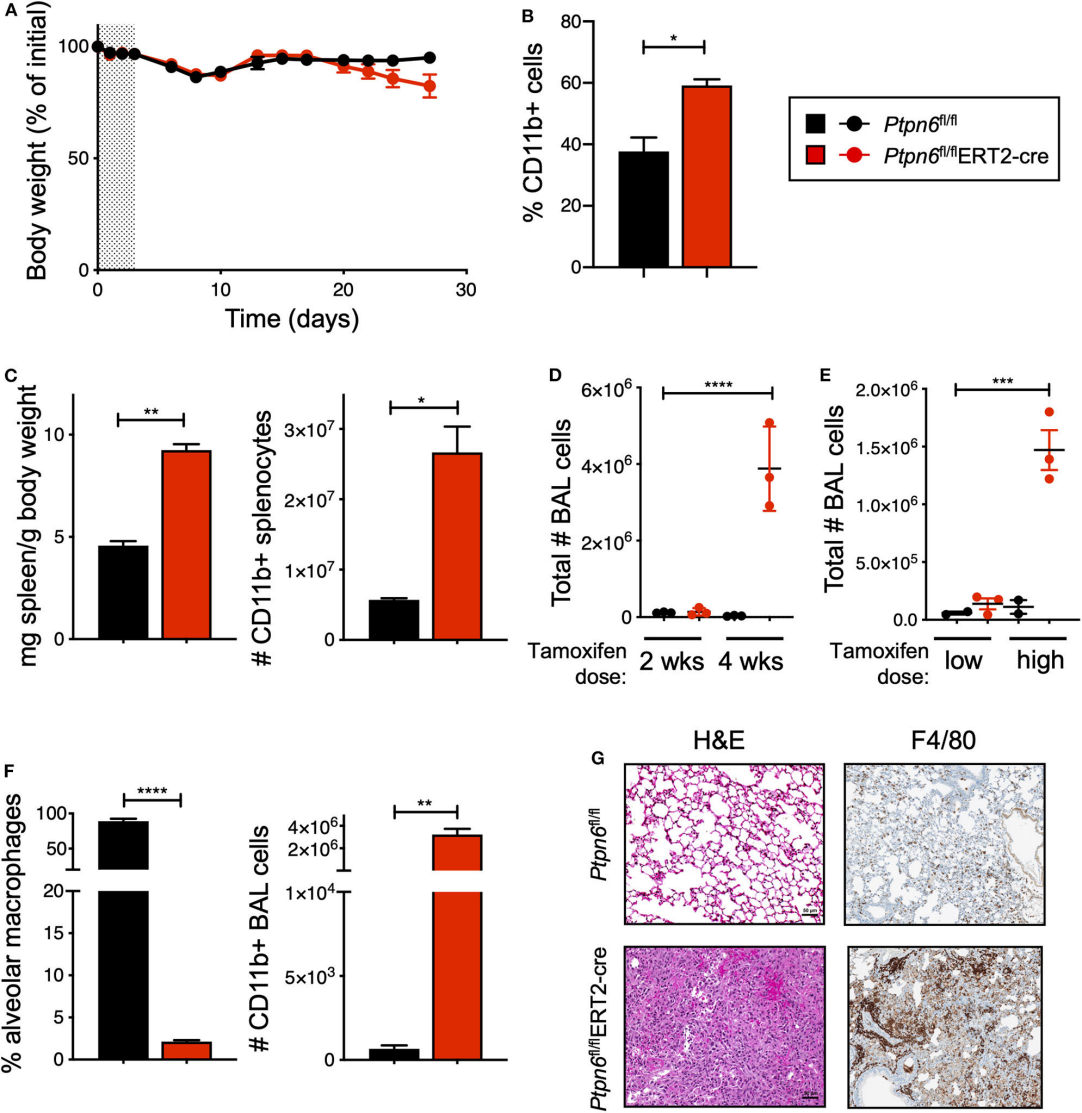
Global, inducible deletion of *Ptpn6* leads to features of the *motheaten* phenotype. **(A)** Body weight of mice of the indicated genotypes, given as % of initial mouse weight, measured over time following treatment with 200 mg/kg tamoxifen bid for 4 days (shaded area). Data is from *n* = 9–10 mice per group. **(B)** Flow cytometric analysis of CD11b^+^ cells in peripheral blood taken 14 days after initial tamoxifen dose, data shown as % of live CD45^+^ cells. **(C)** Spleen weight relative to mouse body weight (left), and flow cytometric analysis of CD11b^+^ splenocytes (right) measured 50 days after initial tamoxifen dose. **(D,E)** total number of bronchoalveolar lavage (BAL) cells from mice of the indicated genotypes measured at either day 14 (2 wks) and 28 (4 wks) following 200 mg/kg tamoxifen bid for 4 days **(D)**, or day 50 following 5 days of 200 mg/kg/day (low dose) or 4 days of 200 mg/kg bid (high dose) **(E)**. **(F)** Flow cytometric analysis of BAL cells at day 28 after initial tamoxifen dose of 200 mg/kg tamoxifen bid for 4 days. Data is graphed as % of CD45^+^ live cells. **(G)** Representative H&E and anti-F4/80-stained sections of lung lobes from mice of the indicated genotypes. H&E stained sections were from lungs harvested 4 weeks after initial tamoxifen dose of 200 mg/kg tamoxifen bid for 4 days, two independent experiments with 4–7 mice/group. Anti-F4/80 staining is from one experiment with 5 mice/group; lungs were collected day 27 after tumor implantation. Error bars represent SEM, *p < 0.05, **p < 0.01, ***p < 0.001, ****p < 0.0001.

Before challenging *Ptpn6* inducible knockout mice with tumors, we wanted to determine the impact of Shp1 loss in non-tumor-bearing mice. In particular, we were intrigued as to whether we would observe the broad immune cell hyper-activation seen in *motheaten* mice using our model, wherein the hematopoietic compartment was allowed to develop normally prior to Shp1 loss. Strikingly, we found that mice with inducible *Ptpn6* deletion developed several features reminiscent of the *motheaten* phenotype. All mice lost weight in the 10 days following tamoxifen administration (Figure 3A), likely as a result of tamoxifen-induced adverse effects (50). Mice recovered weight to baseline within 2 weeks after treatment. However, at day 20, *Ptpn6*^fl/fl^ERT2*-*Cre mice begin to exhibit weight loss (Figure 3A) that was not observed in control *Ptpn6*^fl/fl^ mice. The transient reduction in Shp1 levels did not result in lethal disease as *Ptpn6*^fl/fl^ERT2-cre mice began to regain some weight over time (Supplementary Figure 3A), concomitant with the increase of Shp1 protein in peripheral blood shown in Figure 2A. Expansion of myeloid cells (CD11b^+^) was observed in the peripheral blood of *Ptpn6*^fl/fl^ERT2*-*Cre by day 14 (Figure 3B). We observed splenomegaly in the *Ptpn6*^fl/fl^ERT2*-*Cre mice following tamoxifen treatment, which was due to an increased number of CD11b^+^ myeloid cells (Figure 3C) comprised of both PMNs and Ly6c high and low monocytes (Supplementary Figure 3B). Lung inflammation is a key feature of the *motheaten* phenotype (9); consistent with this, we observed both a time- and dose-dependent increase of total cells within the bronchoalveolar lavage (BAL) fluid of tamoxifen-treated *Ptpn6*^fl/fl^ERT2*-*Cre mice (Figures 3D,E). Whereas BAL fluid harvested from lungs of control mice contained >85% of CD45^+^ CD11c^hi^ alveolar macrophages, the large increase of cells in the BAL fluid from *Ptpn6*^fl/fl^ERT2*-*Cre mice was comprised almost entirely of CD45^+^ CD11b^+^ CD11c^−^ myeloid cells (Figure 3F). Analysis of lung tissue sections from tamoxifen-treated *Ptpn6*^fl/fl^ERT2*-*Cre mice revealed extensive inflammatory cell infiltration (Figure 3G). These data confirmed that deletion of Shp1 in adult mice was sufficient to induce a *motheaten*-like phenotype.

### *Ptpn6* Deletion Drives Robust Anti-Tumor Immunity in Two Immune-Rich Syngeneic Tumor Lines

We next wanted to leverage this novel mouse model to determine the impact of Shp1 loss on tumor growth *in vivo*. We administered tamoxifen according to the regimen outlined in Figure 2A and implanted syngeneic mouse tumor cells 3 days after the final tamoxifen dose, a time selected to coincide with initial Shp1 protein loss in mouse peripheral blood cells. As shown in Figure 2A, reduction in Shp1 protein levels was observed for ~30 days following tamoxifen treatment, which is a sufficient window of time for the syngeneic mouse tumor lines B16F10, E0771, and MC38 to reach endpoint (volume > 2,000 mm^3^). Thus, we reasoned that our model would allow us to interrogate tumor growth co-incident with Shp1 protein loss in the host animals.

**Figure 4:**
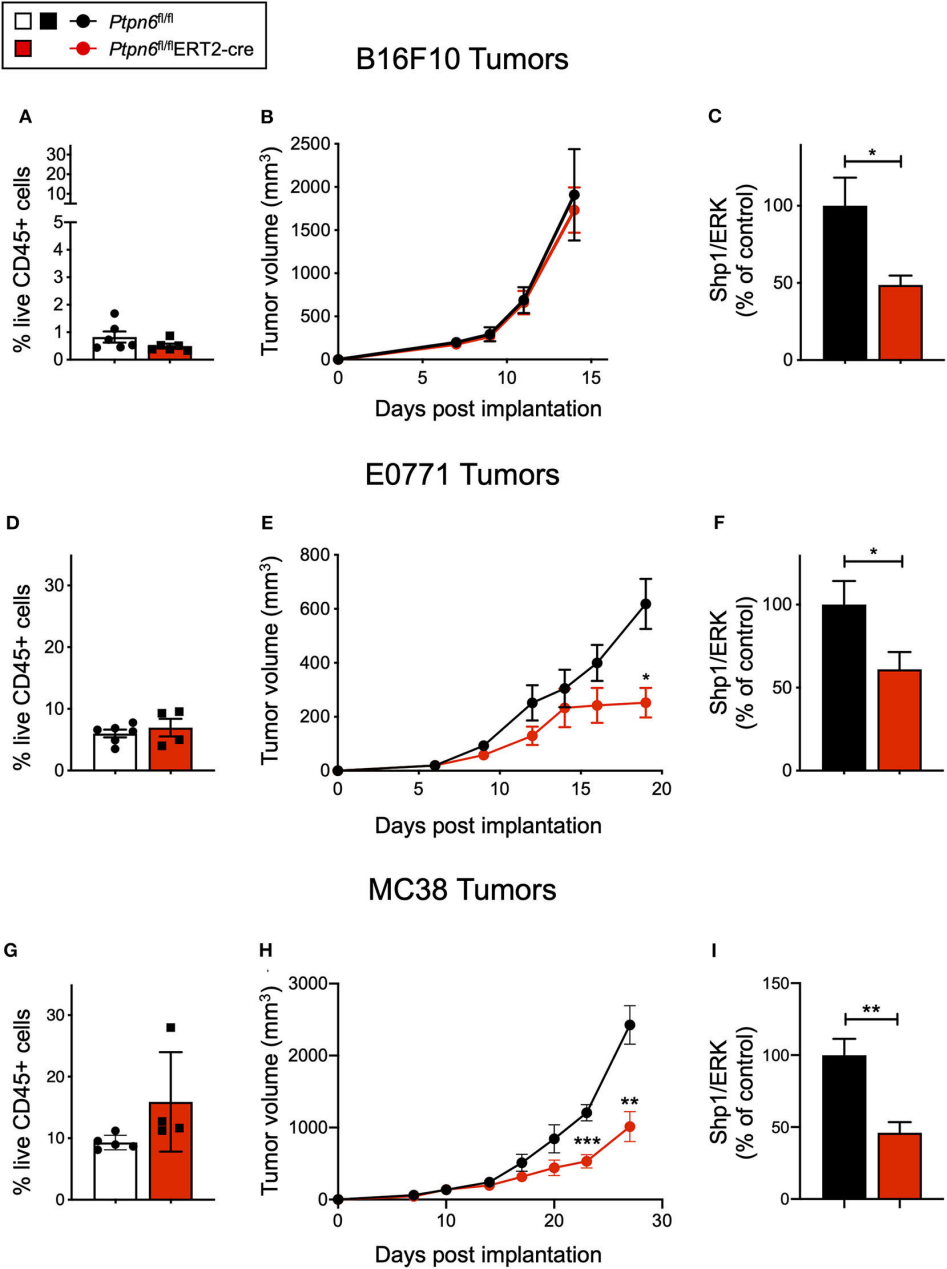
*Ptpn6* deletion drives robust anti-tumor immunity in two immune-rich syngeneic tumor lines. **(A)** Flow cytometric analysis of live CD45^+^ cells from B16F10 melanoma tumors isolated from tamoxifen-treated *Ptpn6*^fl/fl^ and *Ptpn6*^fl/fl^ERT2-cre mice at day 14 post tumor implantation. **(B)** B16F10 tumor volume measurements in tamoxifen-treated *Ptpn6*^fl/fl^ERT2-cre and *Ptpn6*^fl/fl^ mice. Each data point represents the average tumor volume of all mice in a given group. Data is representative of three independent experiments with 5–7 mice/group. **(C)** Shp1 protein relative to total Erk2 protein in peripheral blood cells from indicated mice 14 days after initial tamoxifen dose (200 mg/kg bid for 4 days) and 7 days after B16F10 tumor cells were implanted. Data is representative of at least three independent experiments with 5–7 mice per group. **(D)** Flow cytometric analysis of live CD45^+^ cells from E0771 tumors isolated from tamoxifen-treated *Ptpn6*^fl/fl^ and *Ptpn6*^fl/fl^ERT2-cre mice at day 19 post tumor implantation. **(E)** E0771 tumor volume measurements in tamoxifen-treated *Ptpn6*^fl/fl^ERT2-cre and *Ptpn6*^fl/fl^ mice. Each data point represents the average tumor volume of all mice in a given group. Data is representative of three independent experiments with 4–5 mice per group. Statistical significance was calculated at each time point using an unpaired *t*-test. **(F)** Shp1 protein relative to total Erk2 protein in peripheral blood cells from indicated mice 14 days after initial tamoxifen dose (200 mg/kg bid for 4 days) and 7 days after E0771 tumor cells were implanted. Data is representative of at least three independent experiments with 4–5 mice per group. **(G)** Flow cytometric analysis of live CD45^+^ cells from MC38 tumors isolated from tamoxifen-treated *Ptpn6*^fl/fl^ and *Ptpn6*^fl/fl^ERT2-cre mice at day 29 post tumor implantation. **(H)** MC38 tumor volume measurements in tamoxifen-treated *Ptpn6*^fl/fl^ERT2-cre and *Ptpn6*^fl/fl^ mice. Each data point represents the average tumor volume of all mice in a given group (*n* = 10 *Ptpn6*^fl/fl^ and *n* = 9 *Ptpn6*^fl/fl^ERT2cre, except for days 23 and 27 post tumor implantation, when data collected was from *n* = 9 *Ptpn6*^fl/fl^ and *n* = 7 *Ptpn6*^fl/fl^ERT2-cre/group). Data are representative of two independent experiments with *n* = 9–10 or 4–5 mice per group. Statistical significance was calculated at each time point using an unpaired *t*-test. **(I)** Shp1 protein relative to total Erk2 protein in peripheral blood cells from indicated mice at day 13 post tumor implantation. Mice had been treated with tamoxifen (200 mg/kg bid for 4 days) prior to implantation with MC38 cells. Data is representative of two independent experiments with at least 4 mice/group. Error bars represent SEM, *p < 0.05, **p < 0.01, ***p < 0.001.

B16F10 melanoma is a poorly immunogenic cell line, with <1% of the tumor being comprised of CD45^+^ immune cells (Figure 4A). As such, it is challenging to observe tumor growth inhibition when treating these tumors with single agent immunotherapies (51). Unsurprisingly, we did not observe any difference in tumor growth upon implantation of B16F10 cells into tamoxifen-treated *Ptpn6*^fl/fl^ERT2-Cre and *Ptpn6*^fl/fl^ mice, suggesting that Shp1 loss alone is not sufficient to drive anti-tumor immunity in this “immune desert” tumor (Figure 4B). Immunophenotyping of these tumors did not show any significant changes in the composition of the tumor immune microenvironment (Supplementary Figures 4A,B). All mice reached tumor endpoint by day 15 of the study (Figure 4B) and maintained bodyweight following an initial loss upon tamoxifen administration (Supplementary Figure 4C). We confirmed that Shp1 protein was reduced by 50% in peripheral blood cells of tumor-bearing mice upon tamoxifen treatment (Figure 4C).

Given that Shp1 loss in myeloid cells drives an inflammatory, pro-phagocytic phenotype [(9), Figures 1C–G and Supplementary Figure 1F], we next tested the impact of Shp1 loss on the growth of two syngeneic tumor cell lines that develop a more immune-rich microenvironment, the breast adenocarcinoma E0771 and the colon adenocarcinoma MC38. Myeloid cells made up 80% of tumor-infiltrating leukocytes (CD45^+^ cells) in both E0771 tumors (Supplementary Figure 5A) and MC38 tumors (Supplementary Figure 5B). We did not detect any difference in the total number of live CD45^+^ cells infiltrating the E0771 tumors (~5%) when Shp1 levels were reduced (Figure 4D). However, we observed reduced growth of E0771 tumors in tamoxifen-treated *Ptpn6*^fl/fl^ERT2-cre mice relative to control *Ptpn6*^fl/fl^ mice (Figure 4E), as well as reduced tumor weight (Supplementary Figure 5C), suggesting that loss of Shp1 could drive anti-tumor immunity. We observed a 40% reduction in Shp1 protein levels in peripheral blood cells from tumor-bearing *Ptpn6*^fl/fl^ ERT2-Cre mice following tamoxifen treatment (Figure 4F), and mice began to show bodyweight loss consistent with development of the *motheaten* phenotype (Supplementary Figure 5D).

Analysis of MC38 tumors implanted into our mouse model revealed a higher frequency of CD45^+^ immune cell infiltration (~10–15%) relative to E0771 tumors, but like E0771 we did not observe a significant difference in the overall immune cell infiltrate (% CD45^+^ cells) between mice with reduced Shp1 protein compared to WT controls (Figure 4G). We observed significant MC38 tumor growth inhibition in tamoxifen-treated *Ptpn6*^fl/fl^ERT2-cre mice compared to *Ptpn6*^fl/fl^ control mice (Figure 4H). We confirmed that Shp1 protein was reduced in the peripheral blood of tamoxifen-treated *Ptpn6*^fl/fl^ERT2-cre mice (Figure 4I). We could also detect a 50% reduction of Shp1 protein in MC38 tumor lysates from mice with inducible Shp1 deletion compared to control mice (Supplementary Figure 5E). Given that MC38 tumor cells do not express Shp1 (Supplementary Figure 5E), we attributed the observed reduction in Shp1 protein level to host tumor-infiltrating cells. As expected, MC38 tumor-bearing *Ptpn6*^fl/fl^ERT2-cre mice lost weight, consistent with development of the *motheaten* phenotype (Supplementary Figure 5F).

### Multiple Cell Types Contribute to Anti-Tumor Immunity in Mice With Inducible *Ptpn6* Deletion

**Figure 5:**
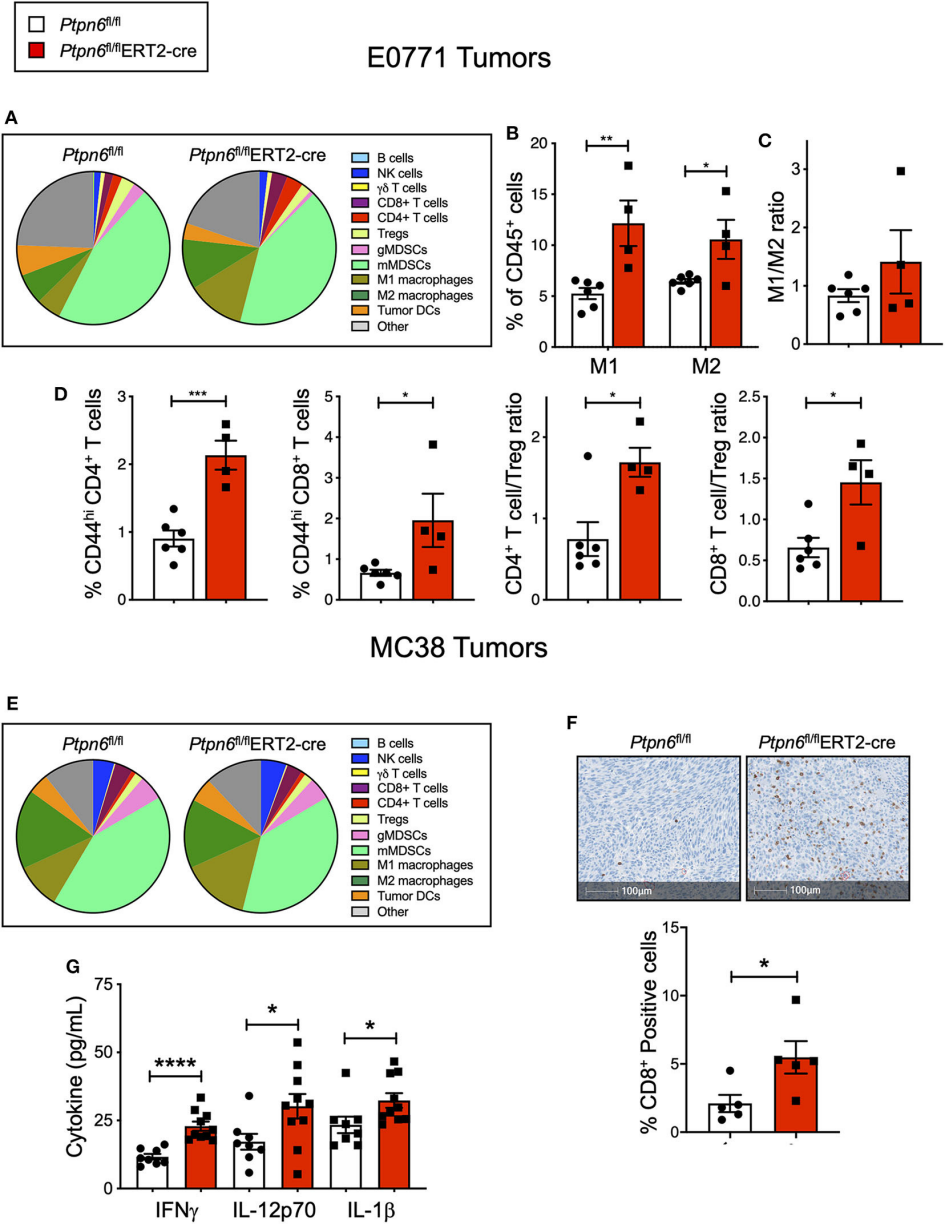
Multiple cell types contribute to anti-tumor immunity in mice with inducible *Ptpn6* deletion. **(A)** Flow cytometric analysis of E0771 tumors isolated from tamoxifen-treated *Ptpn6*^fl/fl^ and *Ptpn6*^fl/fl^ERT2-cre mice at day 19 post tumor implantation. Cells were gated on live CD45^+^ cells and individual populations were identified by the gating strategy outlined in the Methods and Supplementary Figure 8. Data is representative of two experiments with 4–5 mice per group. **(B,C)** Flow cytometric analysis of M1 and M2 macrophages in E0771 tumors from tamoxifen-treated mice, gated based on strategy outlined in Methods and Supplementary Figure 8, represented as % CD45^+^ cells **(B)**, and the ratio of M1 to M2 macrophages **(C)**. **(D)** Flow cytometric analysis of T cell subsets isolated from E0771 tumors from tamoxifen-treated *Ptpn6*^fl/fl^ and *Ptpn6*^fl/fl^ ERT2-cre mice at day 19, represented as %CD45+ cells. Data is representative of two experiments with 4–5 mice per group. **(E)** Flow cytometric analysis of MC38 tumors isolated from tamoxifen-treated *Ptpn6*^fl/fl^ and *Ptpn6*^fl/fl^ERT2-cre mice at day 29 post tumor-implantation. Cells were gated on live CD45^+^ cells and individual populations were identified by the gating strategy outlined in the Methods and Supplementary Figure 8. Data is from one experiment with *n* = 4–5 mice per group. **(F)** Representative image of immunohistochemical analysis of CD8^+^ T cells in MC38 tumors from tamoxifen-treated *Ptpn6*^fl/fl^ and *Ptpn6*^fl/fl^ERT2-cre mice (left), and quantitation of CD8^+^ positive cells in the images (right). Tumors were collected on day 27 post implantation. Data is from one experiment. **(G)** Multiplexed immunoassay (Luminex) analysis of cytokine levels in lysates of MC38 tumor isolated from tamoxifen-treated *Ptpn6*^fl/fl^ and *Ptpn6*^fl/fl^ERT2-cre mice at day 27 post tumor-implantation. Lysates were prepared and analyzed from *n* = 8 *Ptpn6*^fl/fl^ and *n* = 10 *Ptpn6*^fl/fl^ERT2-cre mice. Error bars represent SEM, and *p < 0.05, **p < 0.01, ***p < 0.005, ****p < 0.001.

Immunophenotyping of the E0771 tumors from *Ptpn6*^fl/fl^ERT2-cre and *Ptpn6*^fl/fl^ mice at the study endpoint revealed a significant increase in both the numbers of M1 and M2 macrophages (Figures 5A,B) and a trend toward an increased M1/M2 ratio (Figure 5C). Although we saw no effect on the frequency of αβ T cells (Supplementary Figure 5A), we observed evidence of increased T cell activation: E0771 tumors from *Ptpn6*^fl/fl^ERT2-cre mice contained a higher frequency of activated, antigen-experienced (CD44^hi^) CD4^+^ and CD8^+^ T cells, and an increase in the ratio of effector CD4^+^ and CD8^+^ T cells to Tregs (Figure 5D), consistent with an anti-tumor response.

Immunophenotyping of MC38 tumors collected near study endpoint from tamoxifen-treated *Ptpn6*^fl/fl^ERT2-cre and *Ptpn6*^fl/fl^ mice did not reveal any significant differences in the composition of the immune cell infiltrate (Figure 5E), though there was a trend toward increased M1 macrophages with no effect on the frequency of M2 macrophages (Supplementary Figure 5G). As such, there was a trend toward an increased M1/M2 ratio (Supplementary Figure 5H), though this did not reach statistical significance. Despite not seeing an increase in T cell frequency by flow cytometry (Supplementary Figure 5B) nor any difference in CD8^+^ effector T cell to Treg ratio (Supplementary Figure 5I), immunohistochemistry analysis of tumor sections did reveal a significant increase in CD8^+^ T cells in MC38 tumors from *Ptpn6*^fl/fl^ERT2-cre mice relative to controls. Importantly, the increase in CD8^+^ T cell infiltration was observed in the core of the tumors (Figure 5F). We hypothesized that differences in immune cell function, in addition to immune cell abundance, might be contributing to the MC38 tumor growth inhibition observed in Figure 4H. To address this, we analyzed cytokine levels in MC38 tumor lysates from tamoxifen-treated *Ptpn6*^fl/fl^ERT2-cre and *Ptpn6*^fl/fl^ mice at study endpoint and observed increased levels of the proinflammatory, myeloid-derived cytokines IL-1β, and IL-12p70 (Figure 5G). IL-12p70 production by myeloid cells drives differentiation of T helper 1 (Th1) cells and stimulates production of IFNγ from T and NK cells (52, 53). Consistent with this, we also observed increased IFNγ in MC38 tumor lysates from *Ptpn6*^fl/fl^ERT2-cre mice (Figure 5G). Overall, these data demonstrate that Shp1 loss drives robust anti-tumor immunity against two immune-rich syngeneic tumor lines.

The E0771 and MC38 cell lines we used for these studies were not sensitive to immune checkpoint blockade, as implantation of these cells into wild type mice followed by treatment with anti-PD1 did not affect tumor growth (Supplementary Figures 6A,B). The MC38 line was also insensitive to anti-CD47 treatment (Supplementary Figure 6C), even though the tumor cells did express CD47 at the cell surface (Supplementary Figure 1C). That we were able to see immunomodulation and anti-tumor activity upon Shp1 deletion in these two models is significant given that neither tumor line responded to checkpoint inhibitor blockade, the standard of care immunotherapy treatment. Overall, these results demonstrated that Shp1 loss in the host mouse can impair growth of two distinct immunogenic tumor cell lines *in vivo*, but cannot drive anti-tumor activity in a tumor line that is non-immunogenic (B16F10). Our immunophenotyping data suggest that the observed activity likely comes from effects of Shp1 loss on multiple immune cell types including macrophages and T cells. Furthermore, the contribution of these cells to the anti-tumor response may be distinct among different tumor histotypes.

### Loss of Shp1 Exclusively in the T Cell Compartment Is Insufficient to Drive Anti-Tumor Immunity

We investigated whether loss of Shp1 in the T cell compartment alone was sufficient to cause a reduction in tumor growth. This was achieved by crossing *Ptpn6*^fl/fl^ mice with the T-cell specific distal Lck-Cre strain. T cells from these mice show an increase in the expression of the activation marker CD44, but the mice do not show any sign of inflammation or autoimmune disease (Supplementary Figure 7A) (21). We did not observe any reduction of MC38 tumor growth in these mice (Supplementary Figure 7B). Similar to the *motheaten* mice, mice with loss of Shp1 exclusively in the myeloid compartment (crossing the *Ptpn6*^fl/fl^ mice to the CD11c-Cre strain to target DCs or the MRP8-Cre strain to target neutrophils) results in substantial immune activation in young mice (18), which confounded studies analyzing the immune response to tumors in these animals (unpublished observations). As such, we were unable to assess the effect of *Ptpn6* deletion specifically in myeloid cells on tumor growth. This highlights the need for models that permit cell type-specific inducible deletion, or a specific pharmacological inhibitor of Shp1 that would acutely perturb Shp1 activity in mice.

## Discussion

Enhancing anti-tumor effector functions in immune cells has emerged as a successful therapeutic strategy in cancer. Both innate and adaptive immune cells are present in the tumor immune microenvironment: therapies that target innate cells such as agents that block the CD47-SIRPα interaction are being evaluated in the clinic, and checkpoint inhibitor blockade treatments that promote T cell activity are now FDA-approved for several tumor histotypes (1, 3–5). Enhancing anti-tumor immunity by combining different approaches that engage both innate and adaptive immune cells is thus an attractive strategy. As such, the protein tyrosine phosphatase Shp1 is a promising target owing to its broad hematopoietic expression and its regulation of many immune cell signaling pathways. Here we report a novel mouse model of inducible *Ptpn6* deletion that allowed us to investigate how loss of Shp1 in host immune cells could impact tumor growth. This model demonstrated for the first time that deletion of *Ptpn6* in adult mice drove the same inflammatory phenotype as that seen with constitutive *Ptpn6* deletion. Animals with inducible *Ptpn6* deletion exhibited immune cell infiltration in the spleen, lungs and BAL, along with weight loss. This suggests that the *motheaten* phenotype does not arise from aberrant immune cell development and differentiation.

Loss of Shp1 drove anti-tumor immunity against two independent tumor lines, E0771 and MC38, that induce relatively immune cell-rich tumors containing a high frequency of myeloid cells and are insensitive to anti-PD1 checkpoint inhibitor therapy. We found that Shp1 loss in macrophages enhanced their ability to perform phagocytosis *in vitro*, which could be one mechanism that contributed to the observed increase in anti-tumor immunity following induced deletion of *Ptpn6*. Whether *Ptpn6*-deficient macrophages exhibit increased phagocytosis in the tumor microenvironment, and therefore could increase the activity of anti-tumor antibody therapies remains an exciting open research question. Going forward, it would be important to test this hypothesis in a tumor model responsive to agents that enhance pro-phagocytic signaling via opsonization.

Our *in vivo* data support a mechanism of tumor growth inhibition that involves several immune cell types. We observed evidence of both macrophage expansion and T cell activation, with a shift in the balance of effector vs. regulatory T cells. In addition, we observed increased levels of the myeloid-derived cytokines IL-1β and IL-12p70, which are produced by activated macrophages and DCs. These cytokines promote the differentiation of CD4^+^ T cells toward Th1, and production of IFNγ by cytotoxic innate immune cells (natural killer cells) as well as cytotoxic CD8^+^ T lymphocytes and CD4^+^ Th1 cells (52, 53).

We and others have shown that deletion of Shp1 leads to activation of DCs. DCs from *mev* mice exhibit enhanced secretion of the pro-inflammatory cytokine TNFα in response to stimulation (54), and Shp1-deficient dendritic cells derived from *Ptpn6*^fl/fl^CD11c-Cre mice secrete more of the proinflammatory cytokines IL-6, IL-1β, and TNFα (18, 22). Knocking down *Ptpn6* with shRNA leads to enhanced antigen uptake and priming of T cells, and enhanced activity in an *in vivo* vaccination model of B16F10 melanoma (55). Increased priming of T cells could enhance their response to tumor-specific antigens. Previous studies have also demonstrated an effect of Shp1 loss in T lymphocytes. *Ptpn6*-deficient CD4^+^ and CD8^+^ T cells exhibit increased proliferation (20, 21, 56) and are less sensitive to suppression by regulatory T cells (57). A recent genome-wide CRISPR screen in primary human CD4+ T cells identified *PTPN6* as a negative regulator of T cell proliferation (58). *Ptpn6-*deficient CD8 T cells exhibit enhanced killing, and transfer of *Ptpn6-*deficient CD8 T cells into mice with systemic leukemia improves disease outcome (56). It is attractive to hypothesize that the anti-tumor immunity observed in mice with inducible *Ptpn6* deletion is due in part to enhanced T cell priming by DCs and increased effector T cell activity. Shp1 loss can also impact other cell types that are relevant to tumor immunology, such as B cells, natural killer cells, and neutrophils (18, 25, 26). While we did not detect significant differences in the numbers of these cells in tumors from tamoxifen-treated *Ptpn6*^fl/fl^ERT2-cre mice, it is possible that Shp1 loss alters the activity of these cells in the tumor microenvironment. Examination of other tumor models, that generate different tumor immune microenvironments compared to the models presented in this study, may reveal different responses to genetic deletion of Shp1, either alone or in combination with other therapies.

While our *in vivo* tumor growth data provide a strong rationale for pharmacological inhibition of Shp1 as a potential therapeutic approach for cancer, the development of a *motheaten*-like disease may represent a challenge with respect to tolerability. The current findings highlight the need for Shp1-selective pharmacological agents that can provide transient and reversible inhibition of Shp1 activity, in contrast to the genetic deletion we used in this study, and therefore provide more translatable insights into the tolerability risks. It will be important to determine whether a therapeutic window exists wherein emergent toxicities can be managed or mitigated, while preserving robust anti-tumor activity. If this is the case, then modulation of Shp1 activity with a pharmacological agent represents an attractive immunotherapeutic strategy for the treatment of cancer.

## Data Availability Statement

All datasets presented in this study are included in the article/supplementary material.

## Ethics Statement

The animal studies were reviewed and approved by the Institutional Animal Care and Use Committees (IACUC) of the University of California, San Francisco (UCSF) or Revolution Medicines.

## Author Contributions

DM, CA, EQ, MS, RH, MG, JS, and CL conceptualized the study. DM, CA, DW, AB, AW, CS, TC, TN, NO, and YH performed the experiments. DM, CA, DW, AB, AW, CS, TC, and NO analyzed the data. DM, CA, DW, AB, EQ, JS, and CL contributed to writing. All authors contributed to the article and approved the submitted version.

## Conflict of Interest

DM, DW, AB, CS, TC, TN, NO, MS, RH, MG, EQ, and JS are full time employees and stockholders of Revolution Medicines. The remaining authors declare that the research was conducted in the absence of any commercial or financial relationships that could be construed as a potential conflict of interest.
