# Supplementary material for: Shp1 Loss Enhances Macrophage Effector Function and Promotes Anti-Tumor Immunity
Source: Front Immunol. 2020 Sep 29;11:576310. doi: 10.3389/fimmu.2020.576310 (PMC7550718; doi:10.3389/fimmu.2020.576310)
Supplement: Supplementary file 1 [file Presentation_1.pptx]

## Slide 1
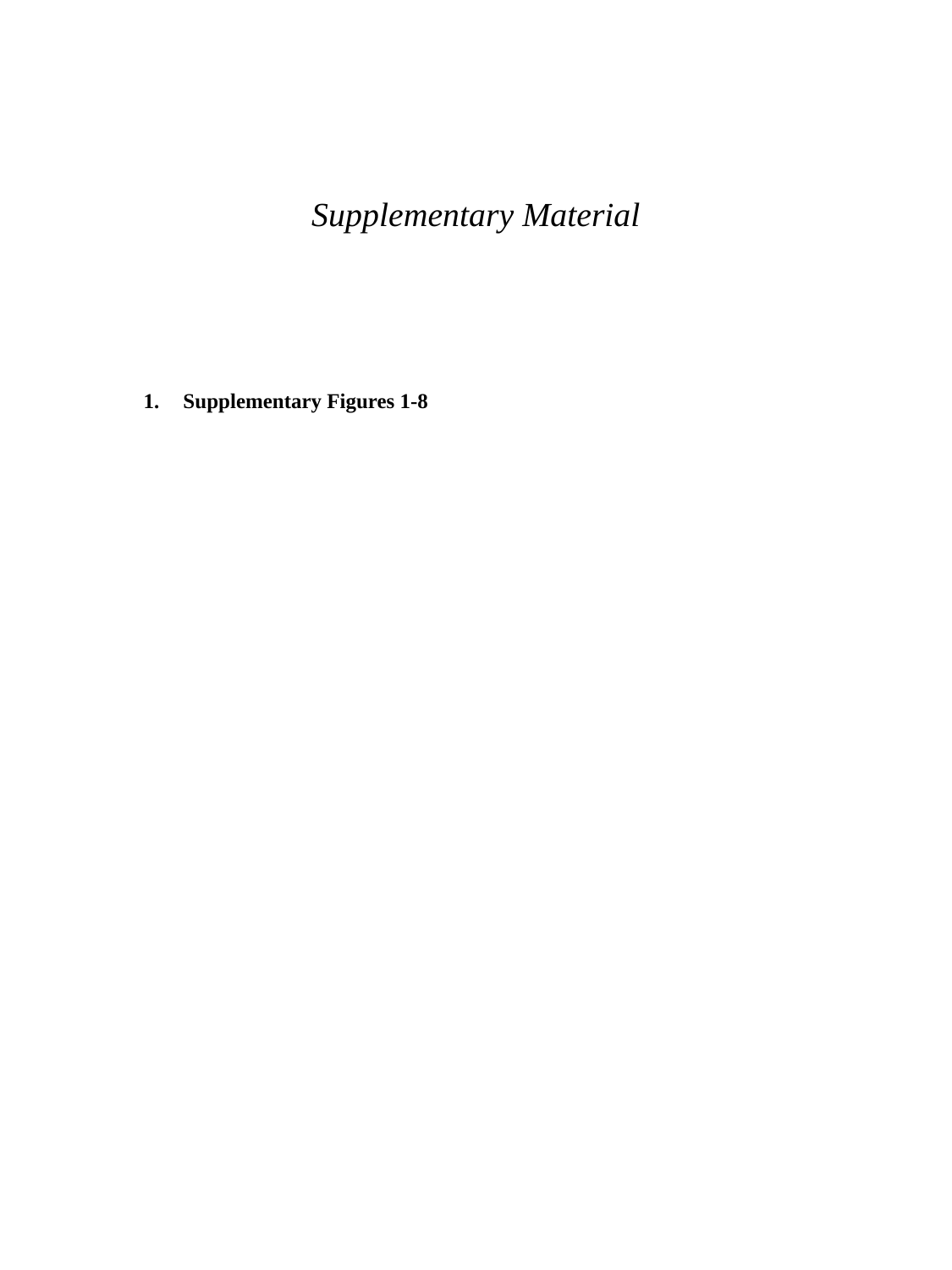

# Supplementary Material
Supplementary Figures 1-8

## Slide 2
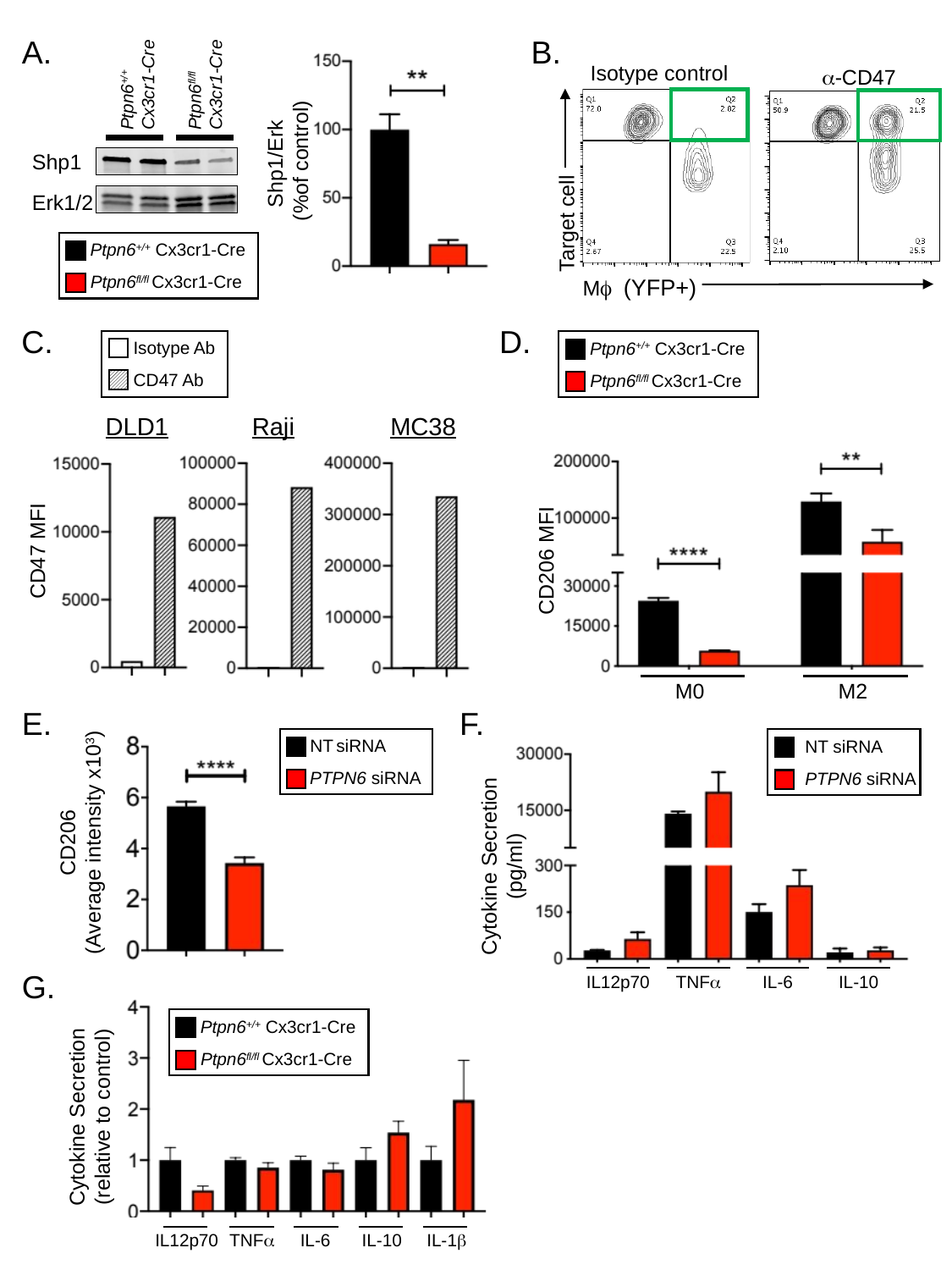

A.
B.
Ptpn6+/+
Cx3cr1-Cre
Ptpn6fl/fl
Cx3cr1-Cre
Shp1
Erk1/2
Shp1/Erk
(%of control)
Isotype control
a-CD47
Target cell
Mf (YFP+)
Ptpn6+/+ Cx3cr1-Cre
Ptpn6fl/fl Cx3cr1-Cre
C.
D.
Isotype Ab
CD47 Ab
Ptpn6+/+ Cx3cr1-Cre
Ptpn6fl/fl Cx3cr1-Cre
DLD1
Raji
MC38
CD206 MFI
M0
M2
CD47 MFI
E.
F.
NT siRNA
PTPN6 siRNA
IL12p70
TNFa
IL-6
IL-10
Cytokine Secretion
(pg/ml)
NT siRNA
PTPN6 siRNA
CD206
(Average intensity x103)
G.
Ptpn6+/+ Cx3cr1-Cre
Ptpn6fl/fl Cx3cr1-Cre
Cytokine Secretion
(relative to control)
IL12p70
TNFa
IL-6
IL-10
IL-1b

## Slide 3
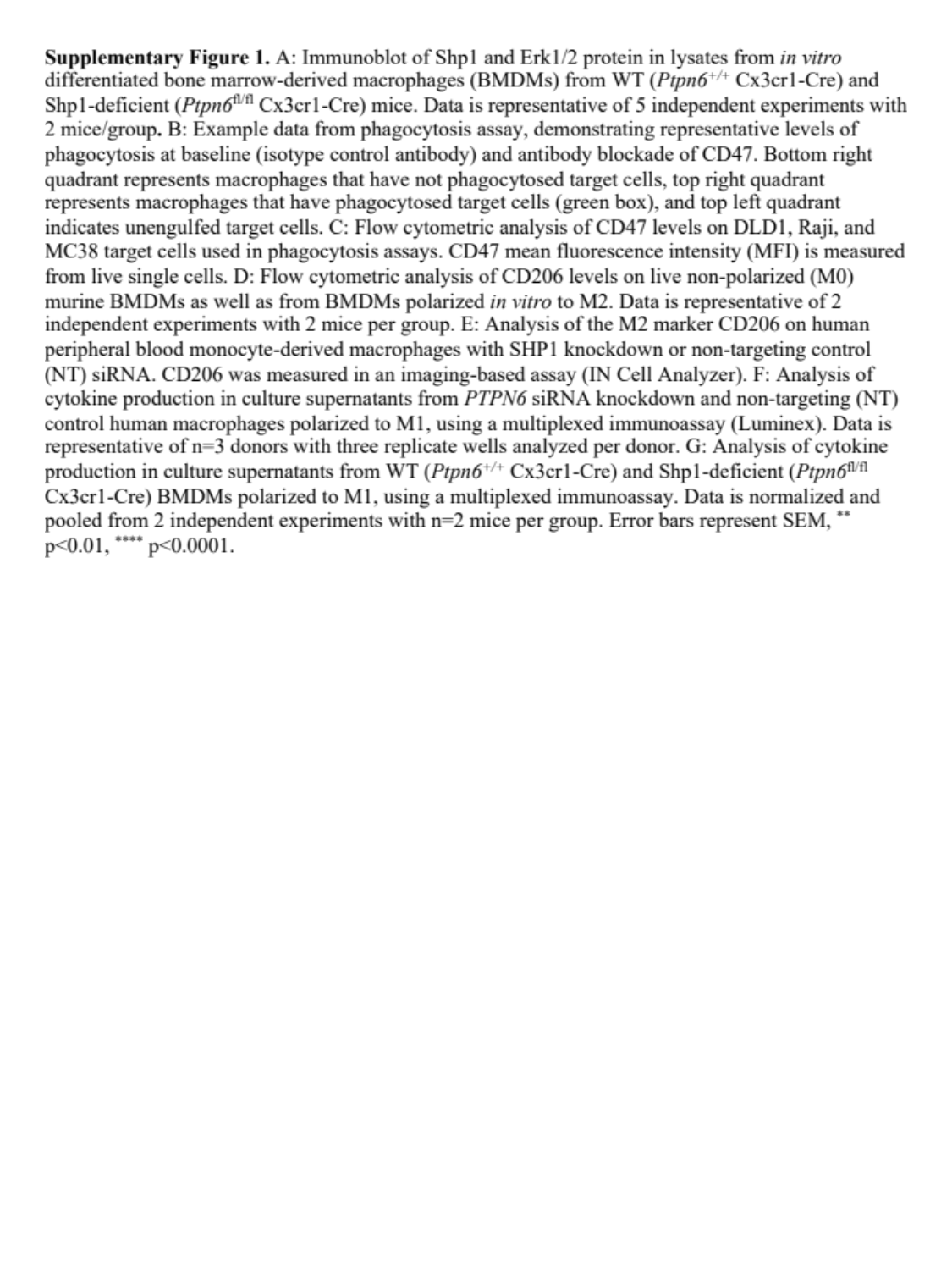

## Slide 4
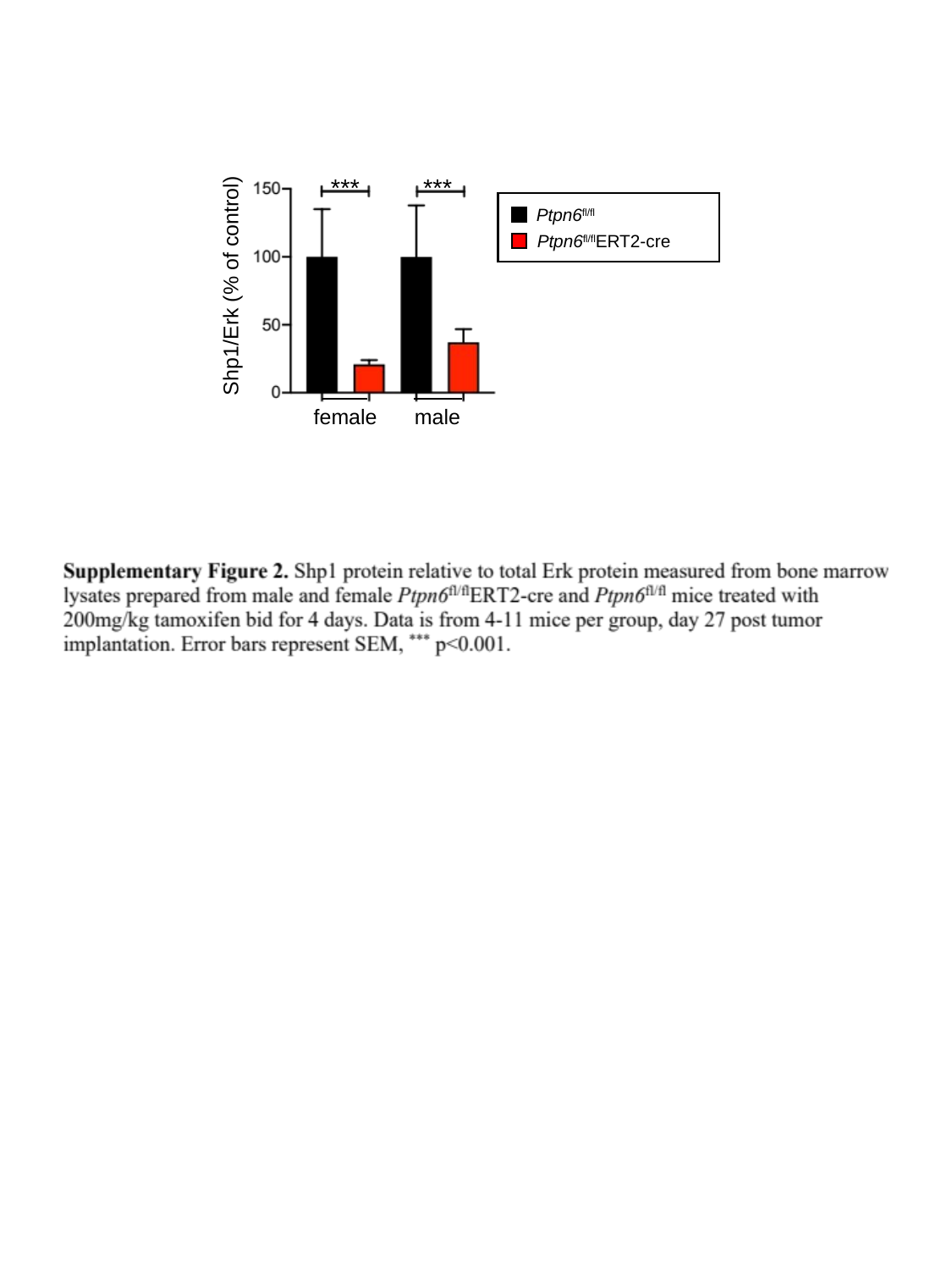

***
***
Ptpn6fl/fl
Ptpn6fl/flERT2-cre
Shp1/Erk (% of control)
female
male

## Slide 5
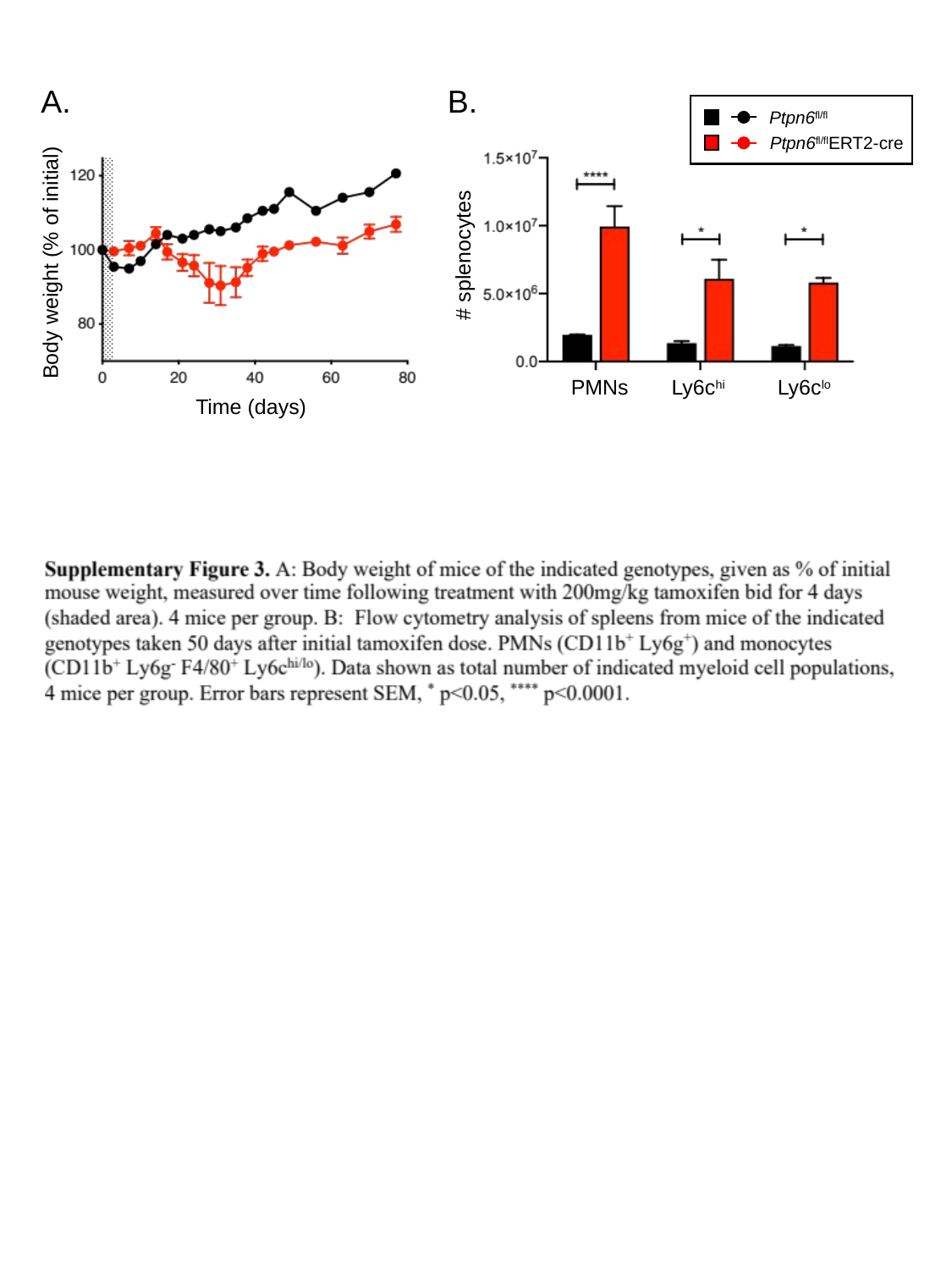

B.
A.
Ptpn6fl/fl
Ptpn6fl/flERT2-cre
# splenocytes
Body weight (% of initial)
PMNs
Ly6chi
Ly6clo
Time (days)

## Slide 6
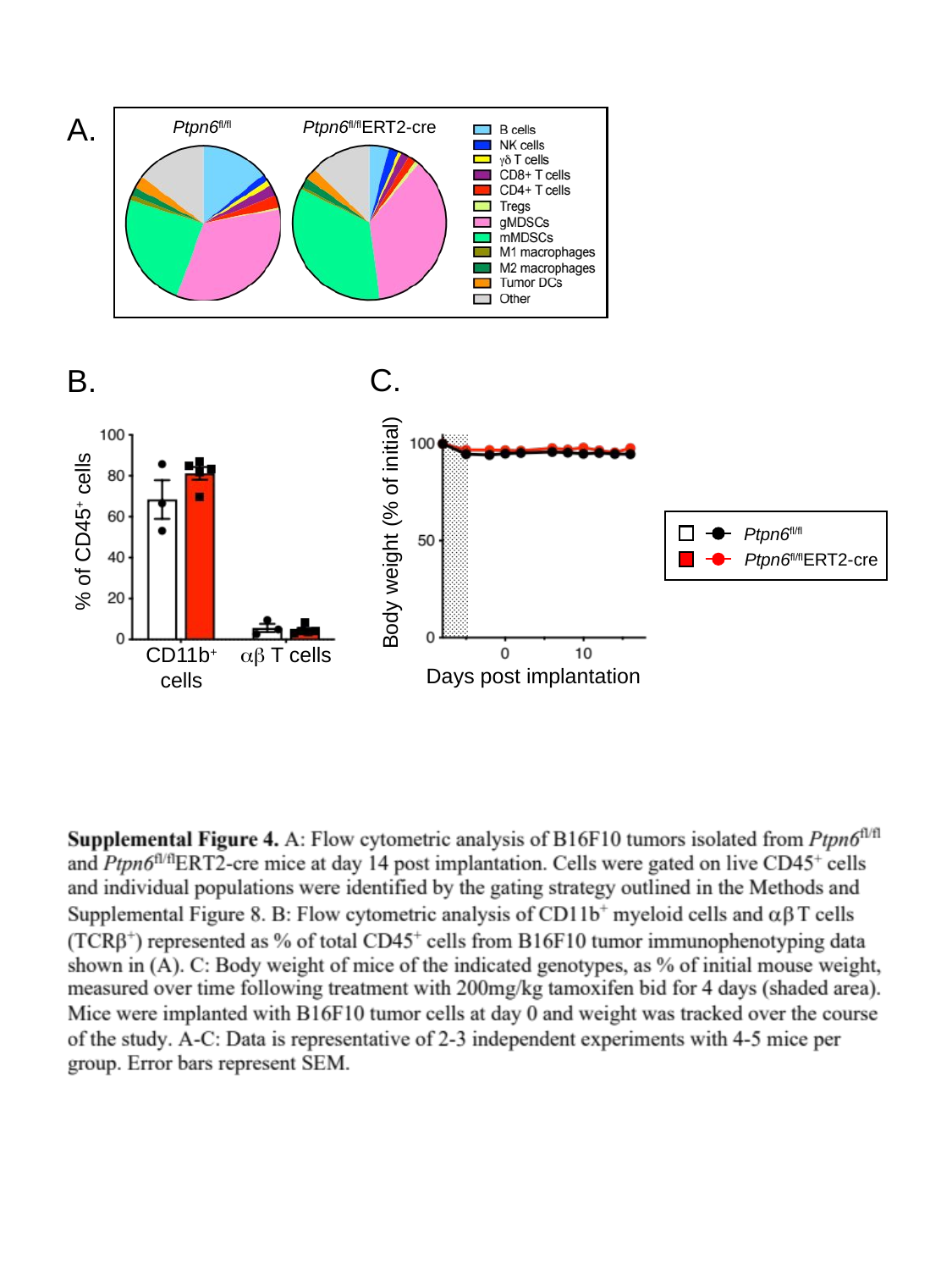

A.
Ptpn6fl/fl
Ptpn6fl/flERT2-cre
C.
B.
Body weight (% of initial)
Days post implantation
% of CD45+ cells
CD11b+
cells
ab T cells
Ptpn6fl/fl
Ptpn6fl/flERT2-cre

## Slide 7
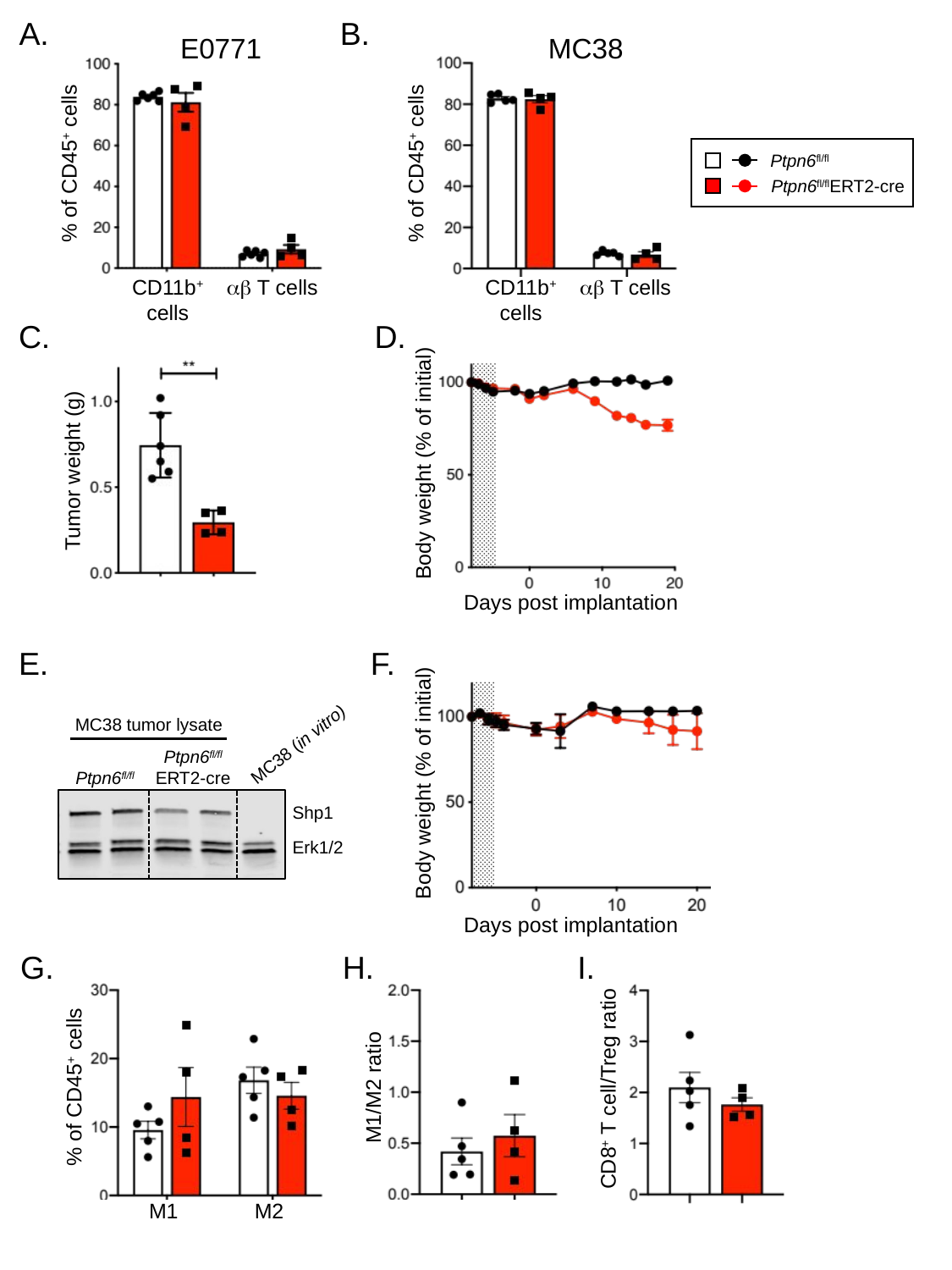

A.
B.
E0771
MC38
Ptpn6fl/fl
Ptpn6fl/flERT2-cre
% of CD45+ cells
% of CD45+ cells
CD11b+
cells
ab T cells
CD11b+
cells
ab T cells
C.
D.
Tumor weight (g)
Body weight (% of initial)
Days post implantation
E.
F.
MC38 tumor lysate
MC38 (in vitro)
Ptpn6fl/fl
ERT2-cre
Ptpn6fl/fl
Body weight (% of initial)
Shp1
Erk1/2
Days post implantation
G.
H.
I.
% of CD45+ cells
M1/M2 ratio
CD8+ T cell/Treg ratio
M1
M2

## Slide 8
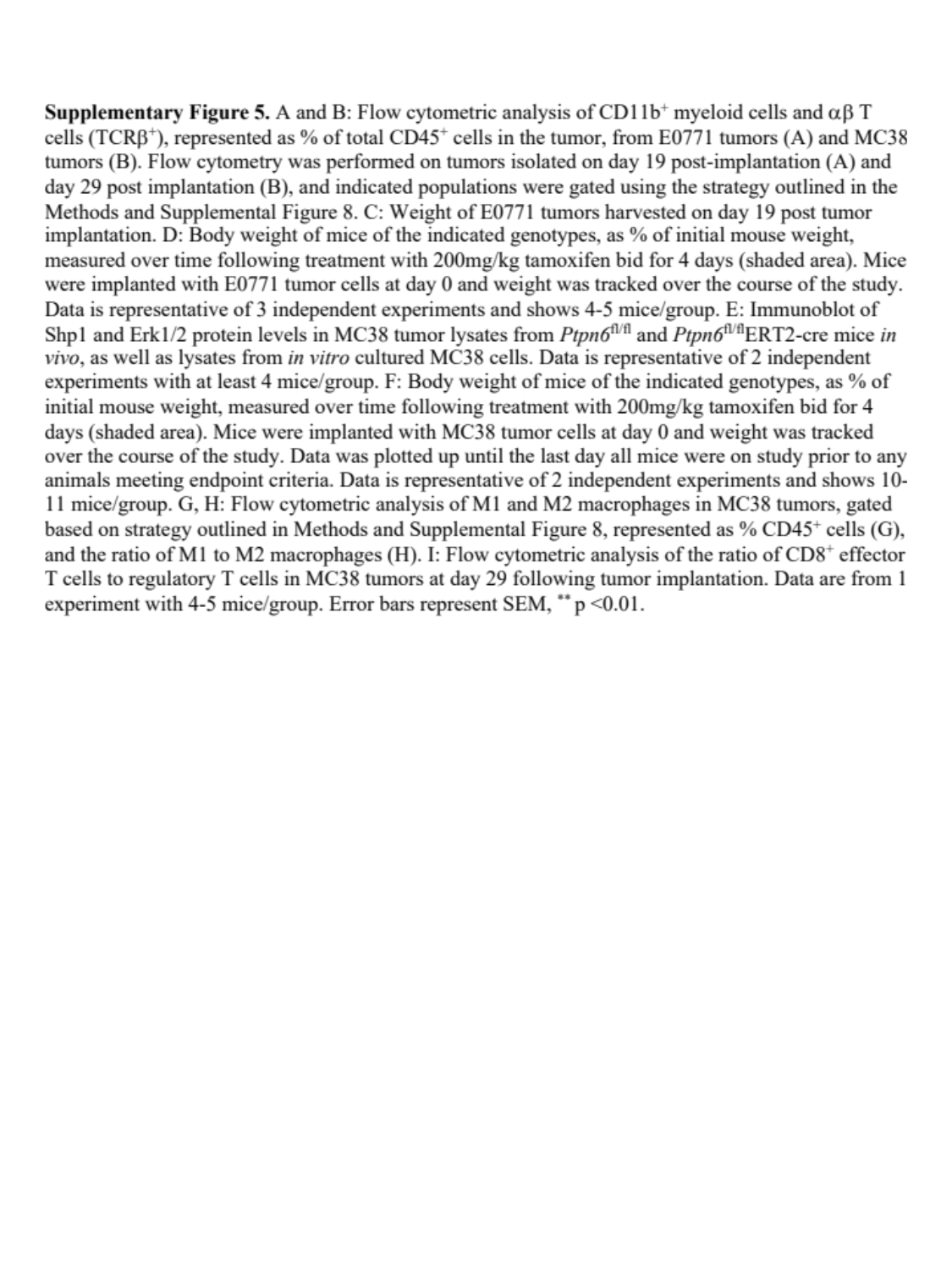

## Slide 9
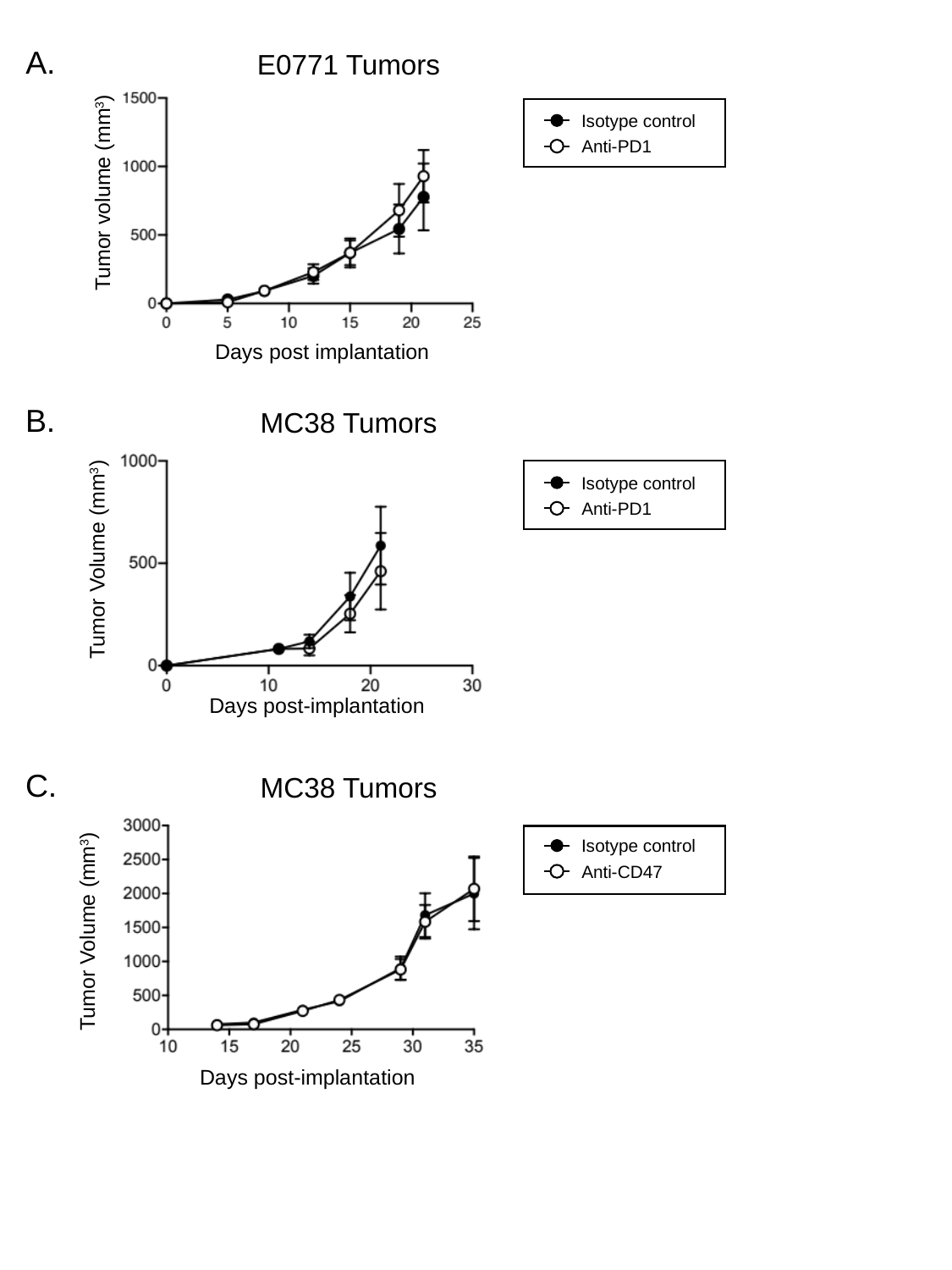

A.
E0771 Tumors
Isotype control
Anti-PD1
Tumor volume (mm3)
Days post implantation
B.
MC38 Tumors
Isotype control
Anti-PD1
Tumor Volume (mm3)
Days post-implantation
C.
MC38 Tumors
Isotype control
Anti-CD47
Tumor Volume (mm3)
Days post-implantation

## Slide 10
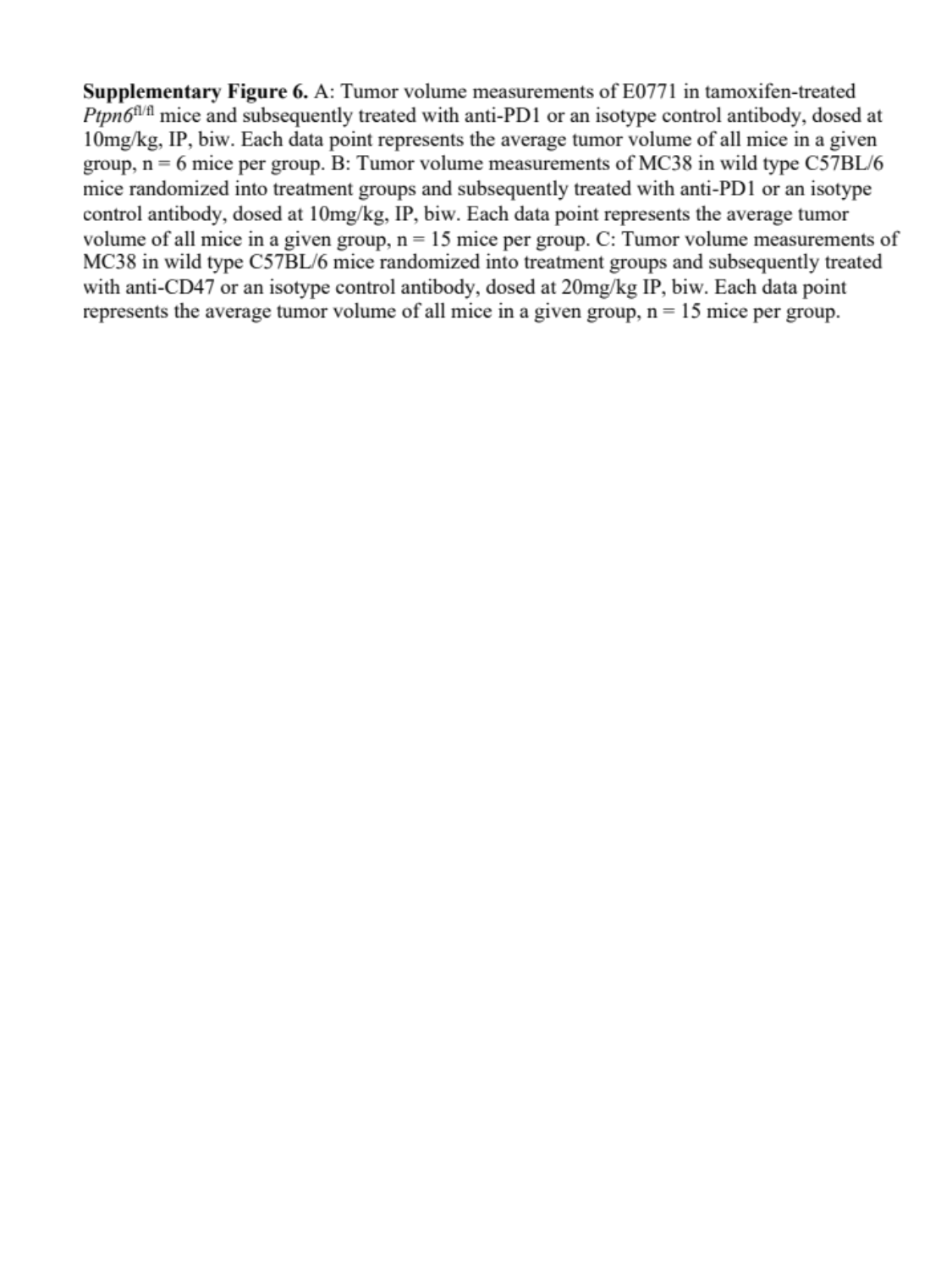

## Slide 11
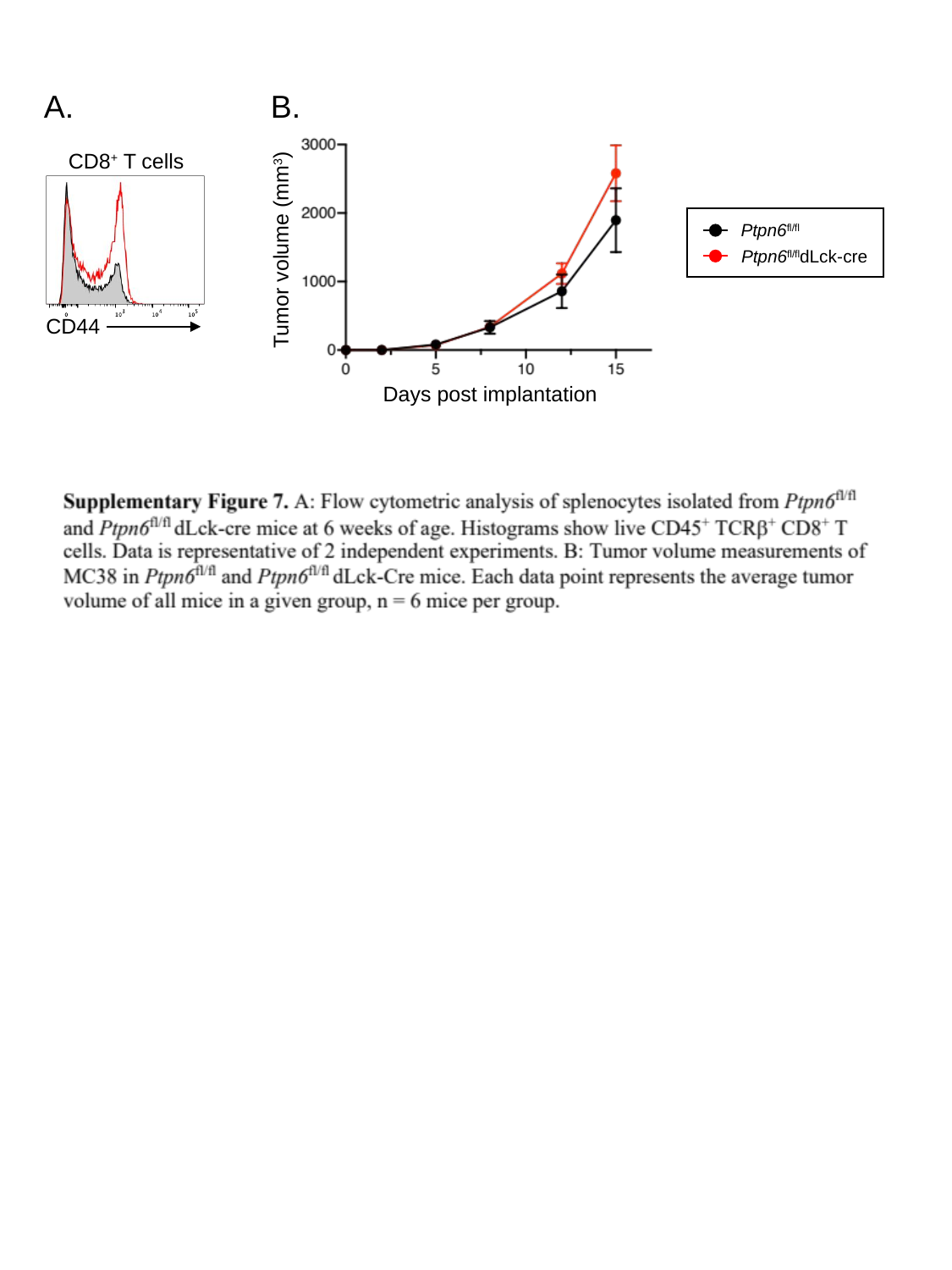

A.
B.
Tumor volume (mm3)
Days post implantation
CD8+ T cells
CD44
Ptpn6fl/fl
Ptpn6fl/fldLck-cre

## Slide 12
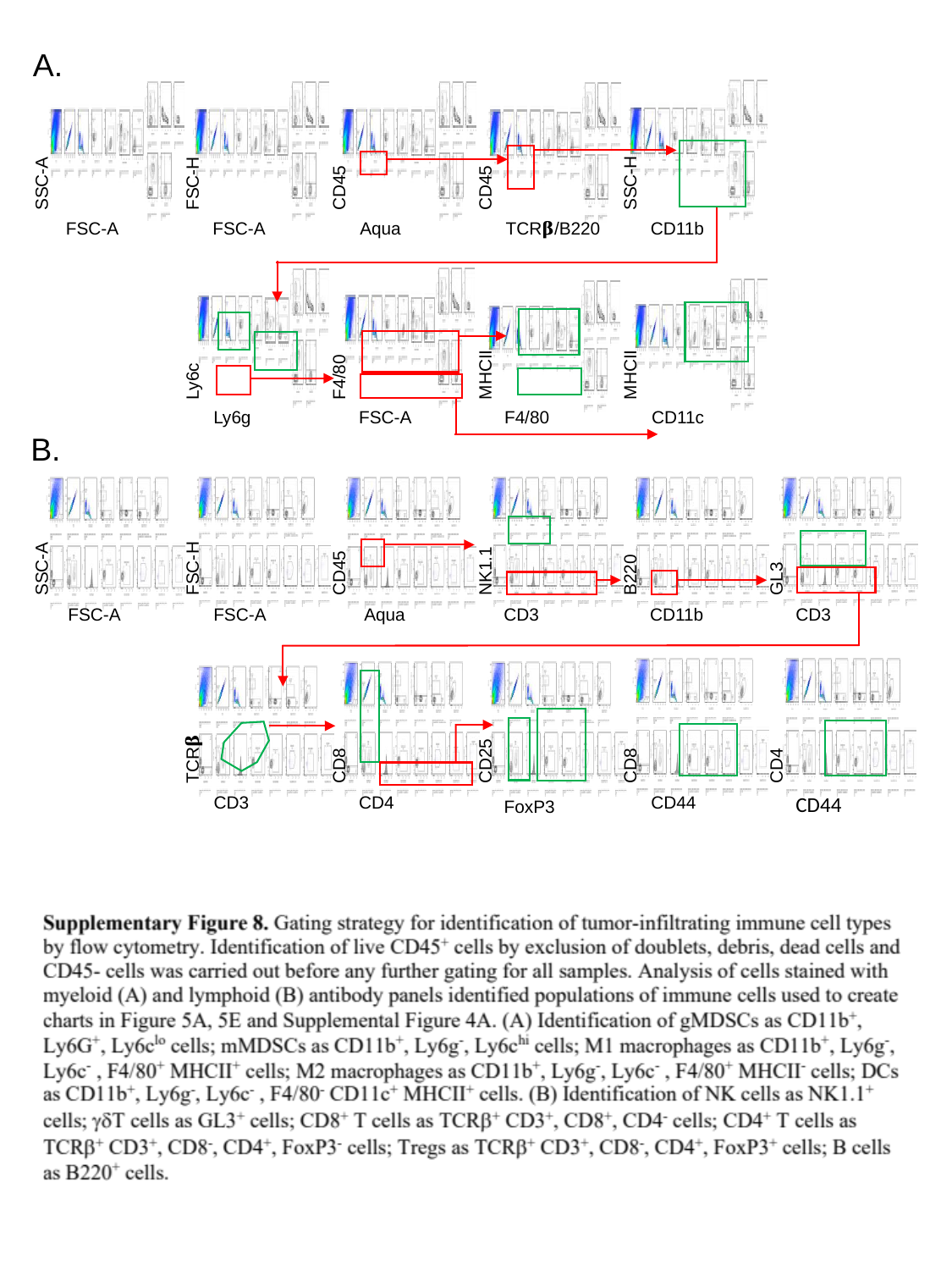

A.
SSC-A
FSC-H
SSC-H
CD45
CD45
FSC-A
FSC-A
Aqua
TCR𝛃/B220
CD11b
MHCII
MHCII
F4/80
Ly6c
Ly6g
FSC-A
F4/80
CD11c
SSC-A
FSC-H
B220
CD45
NK1.1
GL3
FSC-A
FSC-A
Aqua
CD3
CD11b
CD3
CD4
CD8
CD25
CD8
TCR𝛃
CD3
CD4
CD44
CD44
FoxP3
B.
